# Assessing daily energy intake in adult women: validity of a food-recognition mobile application compared to doubly labelled water

**DOI:** 10.3389/fnut.2023.1255499

**Published:** 2023-09-22

**Authors:** Michele Serra, Daniela Alceste, Florian Hauser, Paul J. M. Hulshof, Harro A. J. Meijer, Andreas Thalheimer, Robert E. Steinert, Philipp A. Gerber, Alan C. Spector, Daniel Gero, Marco Bueter

**Affiliations:** ^1^Department of Surgery and Transplantation, University Hospital Zurich, Zurich, Switzerland; ^2^Faculty of Medicine, University of Zurich (UZH), Zurich, Switzerland; ^3^Division of Human Nutrition, Wageningen University, Wageningen, Netherlands; ^4^Centre for Isotope Research (CIO), Energy and Sustainability Research Institute Groningen, University of Groningen, Groningen, Netherlands; ^5^Department of Endocrinology, Diabetology and Clinical Nutrition, University Hospital Zurich, Zurich, Switzerland; ^6^Department of Psychology and Program in Neuroscience, Florida State University, Tallahassee, FL, United States

**Keywords:** dietary intake, energy intake, dietary assessment, mHealth, image-based food recognition, doubly labelled water, artificial intelligence

## Abstract

Accurate dietary assessment is crucial for nutrition and health research. Traditional methods, such as food records, food frequency questionnaires, and 24-hour dietary recalls (24HR), have limitations, such as the need for trained interviewers, time-consuming procedures, and inaccuracies in estimations. Novel technologies, such as image-based dietary assessment apps, have been developed to overcome these limitations. SNAQ is a novel image-based food-recognition app which, based on computer vision, assesses food type and volume, and provides nutritional information about dietary intake. This cross-sectional observational study aimed to investigate the validity of SNAQ as a dietary assessment tool for measuring energy and macronutrient intake in adult women with normal body weight (*n* = 30), compared to doubly labeled water (DLW), a reference method for total daily energy expenditure (TDEE). Energy intake was also estimated using a one-day 24HR for direct comparison. Bland–Altman plots, paired difference tests, and Pearson’s correlation coefficient were used to assess agreement and relationships between the methods. SNAQ showed a slightly higher agreement (bias = −329.6 kcal/day) with DLW for total daily energy intake (TDEI) compared to 24HR (bias = −543.0 kcal/day). While both SNAQ and 24HR tended to underestimate TDEI, only 24HR significantly differed from DLW in this regard (*p* < 0.001). There was no significant relationship between estimated TDEI and TDEE using SNAQ (*R*^2^ = 27%, *p* = 0.50) or 24HR (*R*^2^ = 34%, *p* = 0.20) and there were no significant differences in energy and macronutrient intake estimates between SNAQ and 24HR (Δ = 213.4 kcal/day). In conclusion, these results indicate that SNAQ provides a closer representation of energy intake in adult women with normal body weight than 24HR when compared to DLW, but no relationship was found between the energy estimates of DLW and of the two dietary assessment tools. Further research is needed to determine the clinical relevance and support the implementation of SNAQ in research and clinical settings.

**Clinical trial registration**: This study is registered on ClinicalTrials.gov with the unique identifier NCT04600596 (https://clinicaltrials.gov/ct2/show/NCT04600596).

## 1. Introduction

Accurate assessment of dietary intake plays a crucial role in nutrition and health research, enabling the examination of the relationship between diet and human health (1, 2).

Traditional dietary assessment tools, including food records, food frequency questionnaires, and 24-hour dietary recalls (24HR), have been widely used in nutrition research to capture valuable information on the types and quantities of foods, beverages, and supplements consumed (3–5). One of the most popular and commonly used tools for assessing individuals’ dietary intake in research is the 24HR. This tool, which evaluates an individual nutritional intake over a 24-hour period, can be interviewer-administered or self-administered, providing a relatively simple and participant-friendly approach (6). Notably, the use of 24HR has demonstrated lower misreporting incidence, as well as reduced degrees and variations of underreporting compared to other dietary assessment tools (7).

However, researchers encounter various challenges when measuring energy and nutrient intake in humans. Current dietary assessment tools may require trained interviewers, are time-consuming, and may exhibit limitations in accuracy and qualitative aspects of dietary habits (7–9). Factors such as dieting status, recall bias, social desirability bias, challenges in estimating portion sizes, and omissions of food items can influence the validity of these tools (2, 3, 7). Furthermore, reporting accuracy differs among various populations. Specifically, evidence suggests that individuals with a higher body mass index (BMI) are more likely to underreport their dietary intake (1). Additionally, variations in the accuracy of reporting energy intake have been noted based on sex (7). These findings highlight the importance of considering such factors when assessing self-reported dietary data.

It is crucial to consider not only the reduction of misreporting but also the impact of time-consuming tasks or functions on energy intake when evaluating a dietary assessment tool. Study participants should not be discouraged or inclined to decrease their (reported) energy intake due to the tool’s complexity or time requirements (10, 11). These factors might influence compliance and accuracy, ultimately affecting the validity and reliability of the collected dietary data.

Novel technologies, including web-assisted intake assessments, digital photography, and mobile applications (apps), have been developed to enhance the effectiveness of dietary assessments, aiming to minimize estimation errors, reduce the burden on participants and investigators, and improve the accuracy and efficiency of dietary intake assessments (12–14). These advancements in technology have offered researchers an opportunity to use novel and accurate tools for evaluating dietary intake in real-life settings, contributing to a comprehensive understanding of the impact of diet on overall health and well-being.

Motivated by advancements in information and communication technology, researchers have explored innovative approaches to improve the accuracy of dietary assessment tools (3, 12, 15). Technology-based approaches have gained popularity over conventional tools in the past decade, demonstrating the ability to collect almost real-time data, reduce memory bias, gain user acceptance, and cater to individuals with low literacy levels (4).

*Image-assisted* dietary assessment tools, utilizing wearable cameras or smartphones, complement traditional tools by capturing images of food and beverages to enhance accuracy (16). On the other hand, *image-based* approaches rely primarily on captured images as the main source of information on dietary intake (17). Smartphone apps are used in *image-based* approaches for food recognition and volume and energy estimation, simplifying the recording of dietary intake for both researchers and study participants (18).

Smartphones equipped with advanced features, such as high-resolution cameras, ample memory capacity, strong network capabilities, and faster processors, make them valuable tools for collecting dietary information and capturing food images (19). However, utilizing smartphones for dietary assessment requires training, technical development, secure data transfer infrastructure, and accurate portion size estimations by trained researchers (4). *Image-based* approaches minimize errors from memory recall and portion size estimations (17) and have shown higher estimates of energy intake per eating occasion compared to proxy-assisted records (16), reducing under-reporting when recording food intake with smartphones.

Currently, several apps for *image-based* dietary assessment are available (18), and their use has become feasible due to the widespread ownership of smartphones. A novel *image-based* food-recognition app, SNAQ, utilizes depth-sensing hardware and computer vision to quantify the macronutrient content and quantity of photographed dietary items within the app (20). Designed for real-life scenarios, SNAQ assesses the type and volume of recognized food items, providing information on portion size, macro- and micronutrient intake, food type, eating time, and frequency. Notably, the app is user-friendly and requires less specialized training compared to traditional dietary assessment tools. Its potential as a dietary assessment tool extends to estimating energy, macro-, and micronutrient intake and understanding dietary behavior.

In a previous research work by Herzig et al., it was suggested that SNAQ allows highly accurate volume estimation across a wide range of food items, exhibiting efficient segmentation performance and fast processing time in a controlled setting (20). However, further research is needed to evaluate the validity, accuracy, and precision of these new tools in real-life scenarios (21).

The doubly labeled water (DLW) technique is commonly used to assess the validity of dietary assessment tools by estimating the energy expenditure of an individual during a defined period, usually 7–14 days (22). Based on the assumption of energy balance, hence of body weight stability during the observation period, the energy expenditure estimated with DLW can be compared to the energy intake estimated with the dietary assessment tool under investigation. If the body weight of the individual remains stable during the study period, then the estimated energy intake must equal the energy expenditure. Therefore, any measurement differences between the methods must either be explained by the changes in body weight or be imputed to the dietary assessment tool and the user. This biomarker technique can reveal misreported data of food intake (1, 3).

One approach to assess energy intake reported with a dietary assessment tool is to objectively measure energy intake based on the principle of energy balance by measuring total energy expenditure plus changes in body energy stores, such as fat mass (FM) and fat-free mass (FFM) (22), and to explore the strength of the linear relationship between reported energy intake and body composition (23). Indeed, previous findings suggest that total daily energy intake (TDEI) is proportional to total daily energy expenditure (TDEE) and FFM, but not FM (23–28). These findings suggest not only that energy expenditure itself may have an influence on energy intake (29), but also indicate the presence of a fundamental drive to eat, which serves to meet the energy requirements of vital tissues, organs, and metabolic processes through regular food intake (30–32).

When assessing the validity of an assessment tool for dietary intake, it is important to use multiple statistical tests to gain a comprehensive understanding of its performance. Different facets of validity, such as accuracy, reliability, and agreement, may require different statistical approaches (33). Each of these facets provides distinct information to obtain a more thorough assessment of the performance of the tool. These insights should then be carefully considered when formulating conclusions about the validity of the tool, ensuring they are appropriately aligned with the specific research question and context of the study. In the assessment of validity, it is essential to determine the extent and direction of measurement error, identify factors contributing to such errors, and explore methods to mitigate or address them in the data analyses (33, 34). Furthermore, it is necessary to identify over- and under-reporters by comparing TDEI with TDEE with respect to the basal metabolic rate (Goldberg cut-off points method) before drawing any conclusions regarding validity for a target population (33, 35).

This study aimed to investigate the validity of the SNAQ app as a dietary assessment tool for daily energy intake in adult women with normal weight. The research question was limited to this source population to account for possible confounding of sex and misreporting of individuals with higher BMI. The primary objective was to investigate the agreement between TDEI estimated with the SNAQ app and TDEE estimated with the DLW technique. The secondary objective was to investigate the agreement between TDEI estimated with 24HR and TDEE estimated with DLW. In addition, the agreement between SNAQ and 24HR for TDEI and daily macronutrient intake was also investigated.

## 2. Materials and methods

### 2.1. Setting and study design

A cross-sectional observational study was carried out in 30 adult women with a normal body weight [body mass index (BMI) ≥ 18.5 kg/m^2^ and ≤ 24.9 kg/m^2^] and in free-living conditions. Recruitment and study sessions were conducted between April 2020 and August 2022 at the University Hospital Zurich, Department of Surgery and Transplantation. This investigation is part of a prospective cohort study conducted at the same study site, which explores changes of food intake and selection in patients after Roux-en-Y Gastric Bypass surgery.

The study was conducted according to the Declaration of Helsinki. Ethical approval for this study was received from the Cantonal Ethics Committee of Zurich (BASEC-Nr. 2019-00952) and the study protocol is a secondary analysis of a prospective observational cohort study registered on ClinicalTrials.gov with the unique identifier NCT04600596. Written informed consent was obtained from all the study participants. The results of this study are according to the STROBE-nut statement (36) (Supplementary Table S1).

### 2.2. Participants

Study participants were required to have self-reported literacy in smartphone technology and no history of metabolic and bariatric surgery. Recruitment of study participants was performed at both the University Hospital Zurich and at the University of Zurich, located in Zurich, Switzerland. Exclusion criteria included (1) systemic or gastrointestinal disorders that could impact food consumption or preferences, (2) type 1 or 2 diabetes mellitus, (3) current use of medication or dietary supplements with metabolic and absorptive effects, (4) pregnancy or lactation, (5) adherence to a diet limiting energy intake, (6) renal failure, (7) congestive heart failure, (8) malabsorption syndrome, (9) active and clinically significant psychiatric conditions, such as eating disorders, and (10) inability to comprehend instructions in either German or English (Supplementary Table S2). After inclusion in the study, the study participants received a study identifier and a user account for the study app (see Section 2.7).

The decision to include in this study only adult women with a normal body weight was based on the evidence suggesting that both women and men report different dietary behaviors (7). It remains uncertain whether this is an actual difference or caused by systematic misreporting. Therefore, to avoid any sex-related bias in our results, we decided to exclude men from our study. Furthermore, an increased BMI has been associated with underreporting of energy intake. Therefore, we included only adult women with healthy weight.

### 2.3. Calculation of sample size

The sample size was determined according to the primary outcome of the main study. For this study, using the most conservative assumptions, a difference in energy estimates of 350 ± 450 kcal/day (effect size 0.78) between TDEI reported with SNAQ and TDEE estimated with DLW was considered relevant in assessing the validity of SNAQ (37, 38). Therefore, it was estimated that 27 study participants would have been necessary to have 80% power to detect this difference at a 5% significance level. Accounting for a 10% dropout rate, a sample size of 30 was planned for this study.

In the context of research studies assessing validity of new dietary assessment tools for estimation of TDEI, some authors explicitly refrain from sample size calculation because of recruitment difficulties (39), do not state the calculation of their sample size (40), or simply refer to theoretical papers (41). Therefore, in the absence of a consensus and of thoroughly motivated *a-priori* sample size calculations, it is challenging to define on an individual basis what would be an appropriate accuracy threshold for assessing clinical or research validity of a new dietary assessment tool. Nevertheless, the average sample size of studies assessing the validity of dietary assessment tools by agreement with DLW is 27 (7). In these studies, the sample size was higher when the study design included a laboratory setting (42–44), and it decreased when the study design included free-living conditions (10, 43). Therefore, we considered the calculated sample size (*n* = 30) as appropriate for answering the research question of the main study.

### 2.4. Study outcomes

The primary outcome of this study was the level of agreement between TDEI reported with SNAQ and TDEE estimated with DLW.

The secondary outcomes of this study were the levels of agreement between TDEI reported with 24HR and TDEE estimated with DLW, and the levels of agreement between SNAQ and 24HR for (1) TDEI and (2) macronutrient intake.

Further outcomes were defined to investigated different facets of validity:

1. The strength of linear relationship between TDEE estimated with DLW and TDEI reported with SNAQ and 24HR,
2. The differences of TDEI reported with SNAQ and with 24HR in relation to TDEE estimated with DLW – hereby defined as measurement differences,
3. The comparison, in term of over- and underestimation of energy estimates, of SNAQ and 24HR, to DLW,
4. The classification of over- and under-reporters according to the Goldberg cut-off points,
5. The strength of linear relationship between body composition (FFM and FM) and TDEI reported with SNAQ and 24HR,
6. The strength of linear relationship between body weight changes during the study week and measurement differences of SNAQ and 24HR from DLW,
7. The strength of linear relationship between TDEE during the study week and measurement differences of SNAQ and 24HR from DLW,
8. The extent of stability of diet consumption over the study week,
9. The strength of linear relationship between TDEI reported with SNAQ and TDEI reported with 24HR,
10. The difference in TDEI between estimates of SNAQ and 24HR,
11. The difference in macronutrients intake between estimates of SNAQ and 24HR, and
12. The comparison, in term of over- and underestimation, of SNAQ and 24HR for TDEI and macronutrient intake.

### 2.5. Study protocol

At the screening visit, informed consent was provided by the study participants, inclusion and exclusion criteria were assessed, and data on demographics and anthropometrics were collected. After inclusion in the study, two study visits were set with the study participants at the beginning and at the end of a period of 8 days. This period was necessary to collect data on energy intake and energy expenditure for a period of seven complete days (168 h), where a day was defined as a period of 24 hours. The eight-day period will be hereafter referred to as study week. Participants were instructed to choose a week that reflected their typical daily routines, excluding any exceptional outings or activities.

At the first visit of the study week, the study participants arrived at the study location between 7:00 am and 8:00 am after an instructed overnight fast of at least 8 hours. Baseline anthropometric measurements were performed. The study participants self-reported their dietary habits (whether they adhered to any special diet and which one), smoking habits (whether they smoke or not, independently of the amount and type of cigarettes consumed), and physical activity levels (how many hours per week do they train, independently of the type of activity). Information on food intake in the day immediately preceding the study visit was collected with a 24HR (see Section 2.8). Body composition was measured with a bioelectrical impedance analysis. A pre-dose urine sample was collected. Subsequently, study participants were asked to ingest an individualized dose of DLW (Supplementary Table S3). A second and third urine sample was collected 3 and 4 hours after the dose was ingested, respectively. The overnight fast is observed until the collection of the last urine sample.

On the first day of the study week, before leaving the research facility, the study participants were provided by one of the two study co-investigators with standardized instructions on how to use the app, and a demonstration on how to record food items was given. The study investigator asked the study participant to perform a mock recording. This action was undertaken to verify the capability of the participant to accurately implement the instructions that were provided. Should any difficulties arise during this preliminary phase, supplementary guidance was furnished until the participant exhibited both a comprehensive understanding of the functionality of the app and a proficiency in adhering to the directives provided. In case a study participant did not own a suitable smartphone for the purpose of the study (see Section 2.8), a study smartphone was provided for the duration of the study week. Study participants started reporting their food intake for every eating occasion following the conclusion of the study visit. An “eating occasion” was defined as any occasion where any food or caloric beverage was ingested (45). Three semi-customized prompts were set within the app at the habitual times of breakfast, lunch, and dinner of each study participant. Study participants were also asked to refrain from intense physical activity during the study week or any activities which could cause significant loss of water as sweat. Additionally, study participants were asked not to travel abroad during the study week.

On the eighth day of the study week, study participants returned to the study location for a second study visit before 12 p.m. and did not have to observe an overnight fast. Body weight was measured, and a fourth and fifth urine sample was collected at the same hours of the day as the second and third urine samples, respectively. In case a study participant received a study smartphone, they handed it back at this study visit.

**Figure 1:**
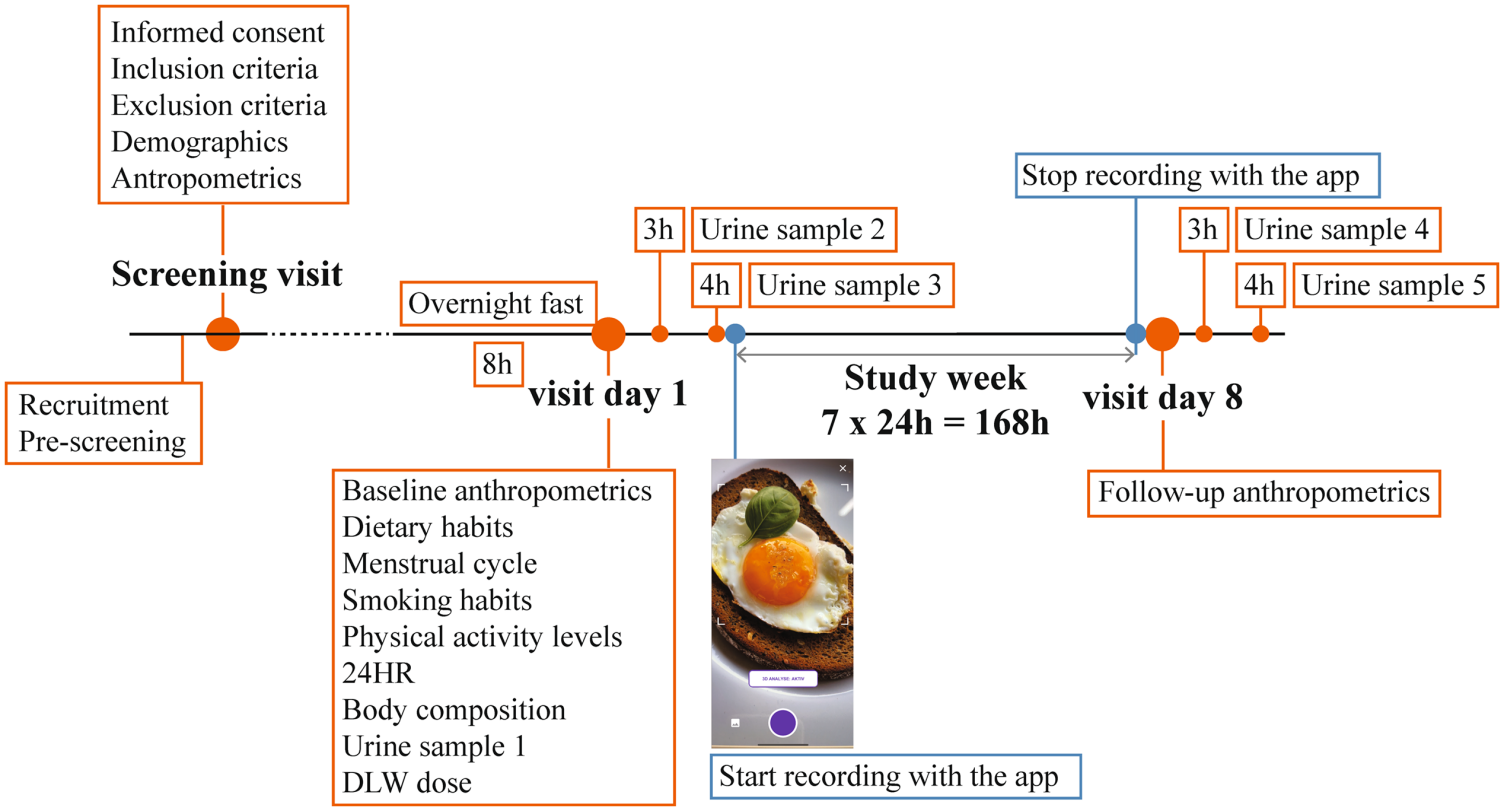
Study design. 24HR, 24-hour dietary recall; DLW, doubly labelled water.

A diagram of the study protocol is shown in Figure 1.

### 2.6. Anthropometric measurements and body composition

Participant height was measured to the nearest 0.01 m with the study subject standing with her back to a wall-mounted stadiometer in bare feet (Seca 274, Seca, Hamburg, Germany). Body weight was measured to the nearest 0.1 kg with a calibrated scale (Seca, Hamburg, Germany). BMI was calculated as weight in kilograms divided by height in squared meters (kg/m^2^). Body composition, including total body water (TBW), fat-free mass (FFM), fat mass (FM), and resting energy expenditure (REE), was measured using a multi-frequency 8-point stand-on bioelectrical impedance device (Seca mBCA 515, Seca, Hamburg, Germany). Additionally, TBW, FFM, and FM were determined by dilution of oxygen-18 (^18^O) and deuterium (^2^H). Isotope dilution spaces (kg) were calculated by the plateau method (46). TBW (kg) was determined by averaging the deuterium dilution space divided by 1.041 and the oxygen dilution space divided by 1.007, with corrections made for *in vivo* isotope exchange (47). FFM (kg) was estimated as TBW divided by 0.732, assuming a hydration factor of 0.732 and considering the body fat to be hydrophobic (48). FM (kg) was estimated by subtracting FFM from the body weight.

The Basal Metabolic Rate (BMR) was calculated with the Mifflin-St Jeor equation for females (49):


$$\begin{array}{l} BMR=\left(10*\mathrm{weight}\;\left(\mathrm{kg}\right)\right)+\left(6.25*\mathrm{height}\;\left(\mathrm{cm}\right)\right)-\\ \;\left(5*\mathrm{age}\;\left(\mathrm{years}\right)\right)-161. \end{array}$$

The Mifflin-St Jeor equation is considered more accurate than the Harris-Benedict equation, and is recommended for calculating BMR in adults (50).

### 2.7. Total daily energy expenditure

Total daily energy expenditure (TDEE) was measured over a seven-day period with the DLW technique (51). The body weight of the study participant was assumed to be stable in the 7-day period of the observation. Briefly, each study participant consumed a dose mixture of 1.8 g of 10 atom% oxygen-18-(^18^O)-labeled water (H_2_^18^O, Cambridge Isotopes Laboratories, Inc. Tewksbury, Massachusetts, United States) per kg body water and of 0.12 g of 99.9 of atom% deuterium-(^2^H)-labeled water (^2^H_2_O, Cambridge Isotopes Laboratories, Inc. Tewksbury, Massachusetts, Unites States) per kg of body water (Supplementary Table S4). Following the “two-point” method protocol described by Schoeller (46, 52), a total of five urine samples were collected for each study participant. One pre-dose and two post-dose urine samples collected at 3 and 4 hours after dosing were collected on the first day. The last two urine samples were collected after 7 days at the same time of the second and third samples, respectively. Isotope analyses of the urine samples were performed at the Centre for Isotope Research (CIO), University of Groningen, Netherlands using an optical spectrometer (LGR LWIA 912–0050, ABB Ltd. – Los Gatos Research, San Jose, California, United States). The measurement analysis (including the sample memory correction) and the calculation of isotopic abundances of ^2^H and ^18^O were done according to the isotope measurement method described by Wang et al. – although in the present study limited to DLW (53). Reference waters (DLW) of the International Atomic Energy Agency (IAEA) were used for calibration (54). Total production of respiratory carbon dioxide (rCO_2_) for calculation of TDEE was calculated according to a new equation proposed by Speakman et al. (55):


$${rCO}_{2}=0.4554*N*\left[\left(1.007*k_{O}\right)-\left(1.043*k_{d}\right)\right]*22.26\text{,}$$

where *N* is the total body water (TBW) estimated using the dilution spaces of both isotopes, and *k_O_* and *k_d_* are the two elimination constants for ^18^O and ^2^H, respectively. TDEE was calculated from rCO_2_ with the Weir equation (56):


$$\mathrm{TDEE}={rCO}_{2}*\left(1.106+\left(\frac{3.94}{RQ}\right)\right)\text{,}$$

where RQ is the respiratory quotient, defined as the proportion between the release of CO_2_ and the O_2_ consumption. TDEE gives an estimation of the daily energy intake under the hypothesis of energy balance, and therefore body weight stability, during the 7 days of the two-point protocol. The use of the coefficients used in the calculations have also been explained by Wang et al. (53).

### 2.8. Dietary assessment with 24HR

Information on dietary intake in the day immediately preceding the study visit was collected by trained study investigators with a 24HR. The study investigators used a standardized pen-and-paper interview, inquiring about all foods, beverages, and supplements consumed in the preceding 24 hours. During the interview, the type of food item was recorded within set eating occasions (breakfast, lunch, dinner, and snacks) together with the approximate self-reported portion size (e.g., tablespoon, cup, plate). Subsequently, the collected recall data were anonymized, transformed into digital format, and processed utilizing a dietary analysis software (Nutritics, version 5.77, 2021, Nutritics, Dublin, Ireland). The software includes food composition data from over 200,000 food items sourced from global food databases, including those from the United States (United States Department of Agriculture), United Kingdom (McCance and Widdowson), and other European countries. It also includes branded food products and raw ingredients. Total daily energy intake (TDEI, in kcal and kJ) and macronutrient intake (carbohydrates in g, fats in g, saturated fats in g, proteins in g, fibers in g, and dietary occasions in *n*) were calculated with Nutritics for each study participant. The estimation of energy intake with the 24HR was not the central focus of this study. Nevertheless, the findings could be instrumental for researchers exploring the congruence between the emergent image-based food-recognition technology and conventional tools.

### 2.9. Dietary assessment with SNAQ

The app *SNAQ study* (SNAQ AG, Zurich, Switzerland), a customized version of the commercial app SNAQ designed specifically for this study, was used by all study participants to self-report their food intake during seven consecutive days. *SNAQ study* was available in two language versions, English and German. Unlike the commercial version of SNAQ, *SNAQ study* features buttons for labeling images of meals as (1) *before* (English version) or *vorher* (German version), which referred to any image taken at the beginning of an eating occasion, (2) *after* (English version) or *nachher* (German version), which referred to any image taken at the end of a eating occasion where the meal was not completely consumed, and (3) *ate everything* (English version) or *alles aufgegessen* (German version), which referred to any image taken at the end of an eating occasion where the meal was completely consumed. Moreover, participants were not granted access to any data pertaining to energy and macronutrient content of the recorded meals which would be normally available on the commercial version of the app. Other attributes of *SNAQ study* were analogue to those of the commercial version. The customized version of the app will hereafter 650 be referred to as app or SNAQ for simplicity.

The food-recognition function of the app is based on computer vision, a type of artificial intelligence for identification and analysis of objects in images. The quality of the results provided by computer vision improves when a depth-sensing camera is used (20). Therefore, following the recommendations of SNAQ AG, only iPhone models X, 11, or 12 (Apple Inc., Cupertino, California), which all include depth-sensing camaras, were selected for this study. For iPhone models X and 11, photographs were taken using the front depth-sensing camera. For iPhone models 12 or above, the back depth-sensing camera was used.

If available, the study participants were asked to install SNAQ study on their personal smartphone. Otherwise, they were provided with a study smartphone (iPhone model 11; Apple Inc., Cupertino, California) solely for the purpose of using SNAQ during the study period.

The study participants were instructed to capture two images (“before,” and “after” or “eat everything”) for each eating occasion. Eating occasions included breakfast, lunch, dinner, snacks, and caloric beverages, except for water and any other energy-free beverages, and supplements. The first image had to be taken prior to consuming the food item(s), while the second image had to be taken at the conclusion of the eating occasion and needed to display any unconsumed food items or the empty plate or glass. Furthermore, in the event of any technical complications or non-adherence to the protocol, participants were instructed to contact a study investigator for instructions. Interactions between the study participants and the investigators were conducted either telephonically or through text messaging, utilizing the personal phone of the participant and the work phone of the study investigator. This communication was permitted at any time to address inquiries specifically related to the study app. Continuous, real-time support was systematically provided by one of the two co-investigators, ensuring availability around the clock to assist with any emergent questions or concerns.

To overcome a potential measurement error of energy intake due to missing inputs of food items the study protocol included the use of semi-customized prompts. Three daily push notifications were set on the study smartphones. The notifications would activate 15 min before three customized times selected by the study participants. These should correspond approximately to the three main eating occasions of the study participants, i.e., breakfast, lunch, and dinner.

**Figure 2:**
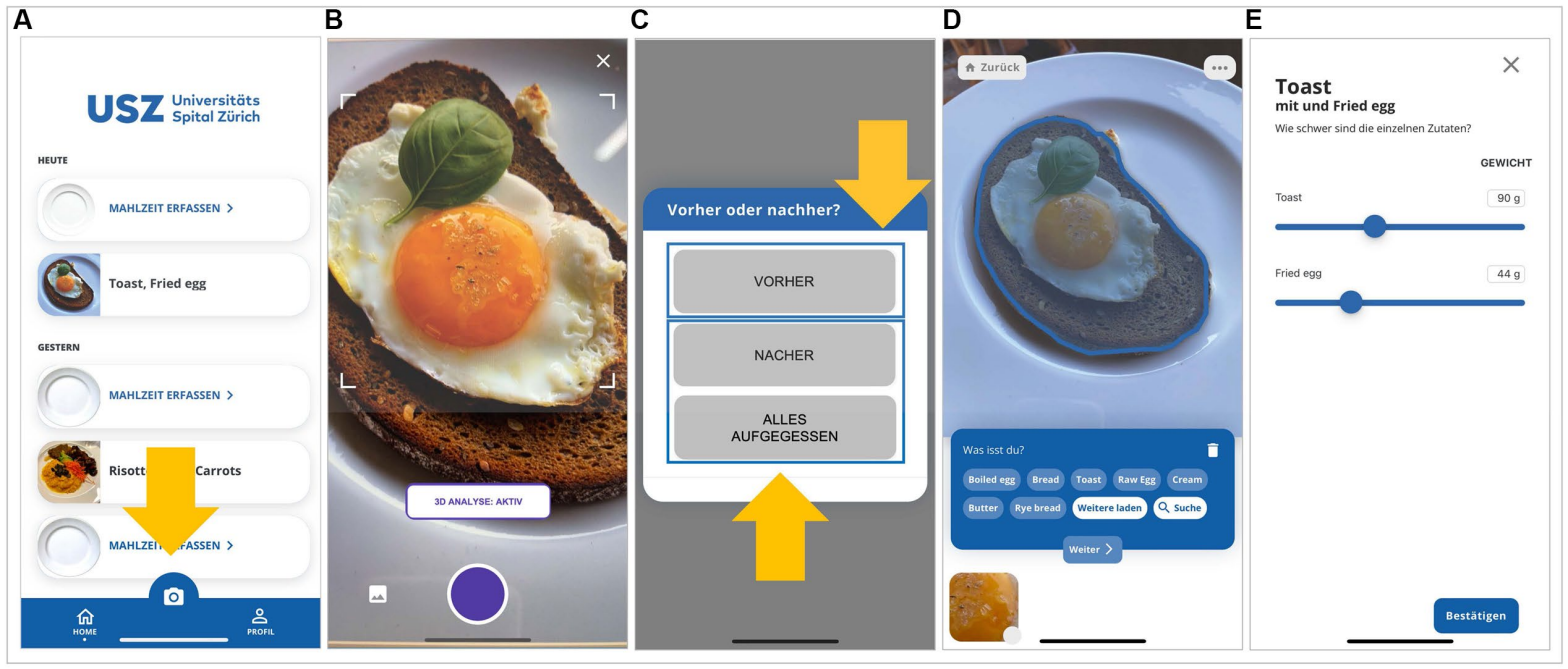
User interface for recording of the eating occasions. **(A)** An image of the food item(s) was captured with the built-in camera app by study participants. **(B)** The food item(s) had to be placed in a white plate or a glass and the image had to be taken from a distance of approximately 30 cm above the item. **(C)** Each image had to be labelled to indicate whether it was taken before or at the end of the dietary occasion. **(D)** If distinct food items were recorded in the image, the computer vision would perform its analysis for these items separately. **(E)** The recognized food type(s) and portion sizes could be manually corrected by the study participants if a food item was not correctly recognized by the app. Translation of the German words in the panels: *alles aufgegessen*, ate everything; *Malzheit erfassen*, register a meal; *nacher*, after; *vorher*, before.

The user interface for recording of the eating occasions is shown in Figure 2. An image of the food item(s) was captured with the built-in camera app by study participants as described above (Figure 2A). The food item(s) had to be placed on a white plate or in a glass and the image had to be taken from a distance of approximately 30 centimeters above the item (Figure 2B). The study participants were instructed to ensure appropriate illumination when the image was taken. If the image appeared blurred, the study participants were instructed to take a photograph with a better quality to improve the performance of the app. Each image had to be labelled, as described above, to indicate whether it was taken before or at the end of the dietary occasion (Figure 2C). The computer vision in the app would then automatically recognize the type and portion size of the food item. If distinct food items were recorded in the image, the computer vision would perform its analysis for these items separately (Figure 2D). The recognized type and portion sizes could be manually corrected by the study participants if the dietary item was not correctly recognized by the app (Figure 2E). The app is designed in such a way that it does not provide the study participants with additional information regarding the macronutrient composition or caloric content of the food items that have been recorded.

Maintaining consistent compliance among study participants for each recording, in accordance with all the instructions delineated for the SNAQ app, presented an insurmountable challenge in a free-living setting. This limitation played a significant role in the decision to utilize DLW as a robust reference method. The concordance between the energy estimates obtained through SNAQ and those derived from DLW served a dual purpose. It not only evaluated the accuracy of the tool itself but also the capability and adherence of the participants as a cohesive entity. In doing so, this comparison effectively assesses the validity of the overall use of the app under the specific conditions defined in this study.

The app utilizes a food density database (SNAQ AG) to transform the estimated food volume into food weight. Then, the app utilizes the Swiss Food Composition Database (57) to estimate the energy content of the recorded food items (20). Given that the computer vision functionality of the app relies on a deep learning model specifically trained on an extensive image catalog of common Swiss foods, the model – at least in the version of the app dedicated to this study – lacks the capability to discern the nutritional values delineated on product labels or to identify product names through natural language processing. Nevertheless, it is important to note that the deep learning model has undergone training with images of packaged food, beverages, and mixed meals. Furthermore, comprehensive nutritional data on a wide array of Swiss food items, encompassing both commonly and rarely commercialized products, is accessible through the Swiss Food Composition Database. Consequently, the information retrieval of the app pertaining to packaged food items is based on this deep learning training, referencing the Swiss Food Composition Database, rather than utilizing natural language recognition techniques.

Data on time of the eating occasions, type of recorded food items, portion sizes, energy and macronutrient content were saved on the servers of SNAQ AG and were extracted at the end of the study for data analysis. Total daily energy intake (TDEI, in kcal and kJ) and macronutrient intake (carbohydrates in g, fats in g, saturated fats in g, proteins in g, fibers in g, and dietary occasions in *n*) were calculated from the extracted data.

### 2.10. Misreporting of total daily energy intake

The misreporting of energy intake was investigated according to the principles of the Goldberg cut-off points (58) following the approach proposed by Black (35). The cut-off points were used for the classification of study participant to comparative purposes with other studies and not for the detection of outliers to be excluded from the data analysis. Under-reporters were defined as having a TDEI:BMR ratio smaller than the lower 95% confidence limit, and over-reporters were defined as having a TDEI:BMR ratio greater than the upper 95% confidence limit. The following two relationships were assessed for the 30 study participants considered in this study with the values of TDEI reported with SNAQ and 24HR:


$$\frac{\mathrm{TDEI}}{BMR}>\mathrm{PAL}\times e^{\left[s.d._{\text{min}}\times \frac{\frac{S}{100}}{\sqrt{n}}\right]}\text{,}$$

$$\frac{\mathrm{TDEI}}{BMR}<\mathrm{PAL}\times e^{\left[s.d._{\text{max}}\times \frac{\frac{S}{100}}{\sqrt{n}}\right]}$$

where BMR is the basal metabolic rate, *PAL* is the mean physical activity level for the study population, s.d._min_ is −2 for the 95% lower confidence limit, s.d._max_ is +2 for the 95% upper confidence limit, and *n* is the number of study participants. BMR was calculated with the Mifflin-St Jeor equation. PAL was calculated as TDEE:BMR ratio. *S* is the factor that incorporates the variation in energy intake, BMR, and energy requirements, and was determined by:


$$S=\sqrt{\frac{{CV}_{\mathrm{wTDEI}}^{2}}{d}+{CV}_{wB}^{2}+{CV}_{tP}^{2}}\text{,}$$

where CV_wTDEI_ is the mean within-subject coefficient of variation for TDEI reported with SNAQ, *d* is the number of days of dietary assessment, CV_wB_ is the coefficient of variation of repeated BMR measurements, and CV_tP_ is the total variation in PAL. CV_wTDEI_ was calculated for this study only for TDEI reported with SNAQ. As far as regard TDEI reported with 24HR, no repeated measurements were available according to the present study protocol. For this study, two values of CV_wTDEI_ and of CV_tP_ were used for the calculation of S: (1) the value of CV_wTDEI_ and CV_tP_ specific for this study population, and (2) the value of CV_wTDEI_ of 26% and the value of CV_tP_ of 16.5% suggested by Black for adult women with normal weight (35). This value of CV_tP_ was suggested for women between 18 and 29 years of age and a mean PAL of 1.70 (35). As far as regards CV_wB_, the coefficient 4.1% was used. This value of CV_wB_ was suggested for women in free-living conditions (59).

The CV_wTDEI_ has been calculated for this study according to the formula (35):


$${CV}_{\mathrm{wTDEI}}=\sqrt{\overset{n}{\underset{i=1}{\sum }}\frac{{CV}_{i}^{2}}{n}}\text{,}$$

where CV*_i_* is the coefficient of variation (CV) calculated for each study participant from the number of days of dietary assessment with SNAQ available for that study participant, and *n* is the number of study participants. The CV*_i_* was calculated by dividing the standard deviation (SD) of the variable of interest by the mean for each study participant. The result was multiplied by 100 to express the CV as a percentage. This calculation provided a measure of the variability within the data of each study participant, expressed as a percentage of the mean value. It is commonly used to assess the reliability or consistency of measurements within individuals. CV_tP_, being a coefficient of variation, was calculated with the same approach of the CV*_i_* of CV_wTDEI_.

### 2.11. Statistical analysis

Data analysis was performed using R (version 4.2.2) (60) through RStudio (version 2022.07.1) (61). Barplots, boxplots, scatter plots, violin plots, and Bland–Altman plots (Figures 3–7 and Supplementary Figures S1–S7) were generated with the ggplot2 R package (version 3.4.0). All figures were organized using Adobe Illustrator (version 27.4.1).

**Figure 3:**
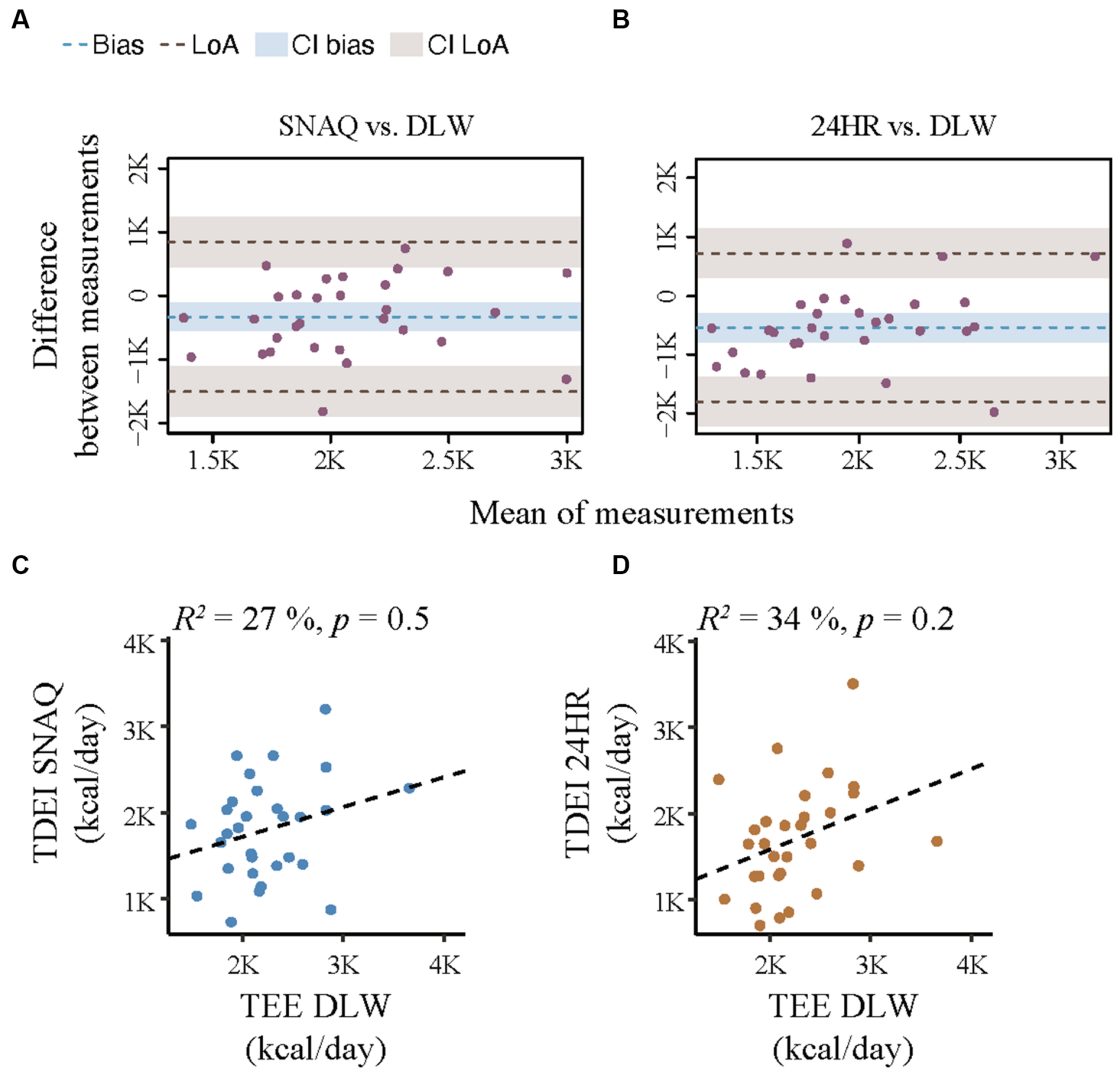
Bland–Altman plots for agreement of the SNAQ app and the 24-hour dietary recall (24HR) with the doubly labelled water (DLW) technique and correlation analysis of linear relationship between energy expenditure and the energy intake estimated with SNAQ and 24HR. **(A)** Bland–Altman plot for agreement on energy intake between SNAQ and DLW. **(B)** Bland–Altman plot for agreement on energy intake between 24HR and DLW. The bias is represented as a blue horizontal dotted line. The value of the bias is estimated by the mean difference in energy estimation between DLW and SNAQ or 24HR. The 95% limits of agreement (LoA) are represented as two brown dotted lines and are defined as mean difference ± 1.96 standard deviations. **(C)** Linear correlation between energy estimates of SNAQ and DLW. **(D)** Linear correlation between energy estimates of 24HR and DLW. 24HR, 24-hour dietary recall; CI, confidence interval; DLW, doubly labelled water; K, decimal unit suffix for thousand; LoA, limit of agreement, *p*, *p*-value of the coefficient of determination; *R*^2^, coefficient of determination of the linear relationship.

**Figure 4:**
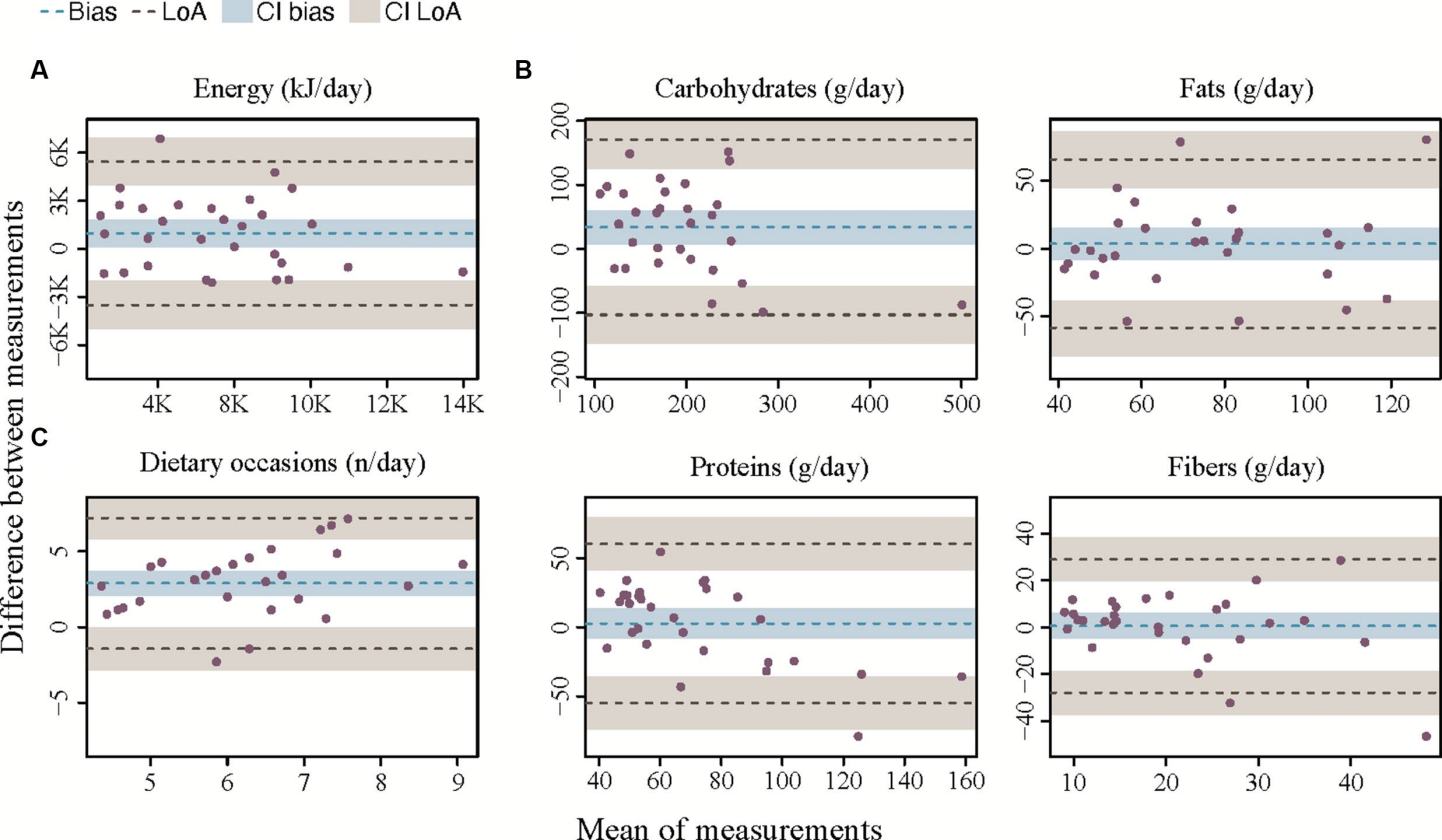
Bland–Altman plots for agreement between the app and the 24HR. **(A)** Bland–Altman plot for total daily energy intake. **(B)** Bland–Altman plots for macronutrient intake. **(C)** Bland–Altman plot for number of eating occasions. The bias is represented as a black horizontal line. The value of the bias is estimated by the mean difference in intake estimation between 24HR and the app for total daily energy intake and macronutrient intake. The 95% limits of agreement (LoA) are represented as two dotted lines and are defined as mean difference ± 1.96 standard deviations.

**Figure 5:**
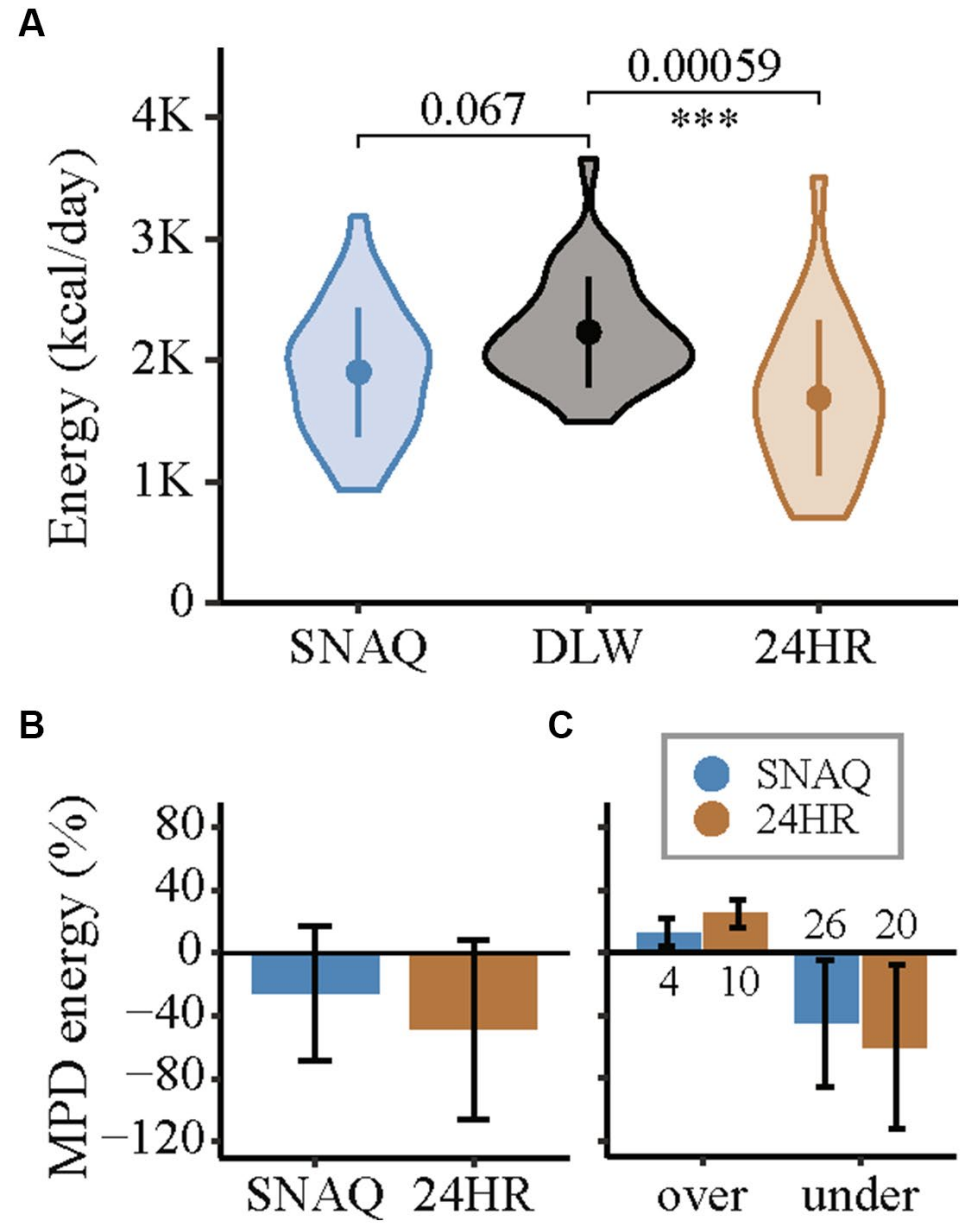
Measurement differences of total daily energy intake of the app SNAQ and the 24-hour dietary recall (24HR) in relation to the total daily energy expenditure estimated with the doubly labelled water (DLW) technique. **(A)** Violin plots of the energy estimates of SNAQ, DLW, and 24HR. Mean of the groups are represented with full points in the middle of the violin plots. Standard deviations are represented with a vertical line in the middle of the violin plots. Pairwise comparisons of SNAQ and DLW, and 24HR and DLW are represented with horizontal lines between the violin plots. *p*-values of the pairwise comparisons are reported above the horizontal lines. Significance levels were adjusted for multiple comparison (3x) and are reported below the horizontal lines. They are expressed as follows: *when *p*-value < 0.05; **when *p*-value < 0.01; *** when *p*-value < 0.001. **(B)** Mean percentage differences for estimations of daily energy intake of SNAQ and 24HR in relation to DLW. **(C)** Mean percentage differences for over- and underestimations of total daily energy intake of SNAQ and 24HR in relation to total daily energy expenditure estimated by DLW. The number of over- and underestimations between study participants are reported as numbers below the bars, for overestimations, or above, for underestimation. 24HR, 24-hour dietary recall; DLW, doubly labelled water; *K*, decimal unit suffix for thousand; MPD, mean percentage difference; over, overestimation of SNAQ and 24HR in relation to DLW; under, underestimation of SNAQ and 24HR in relation to DLW.

**Figure 6:**
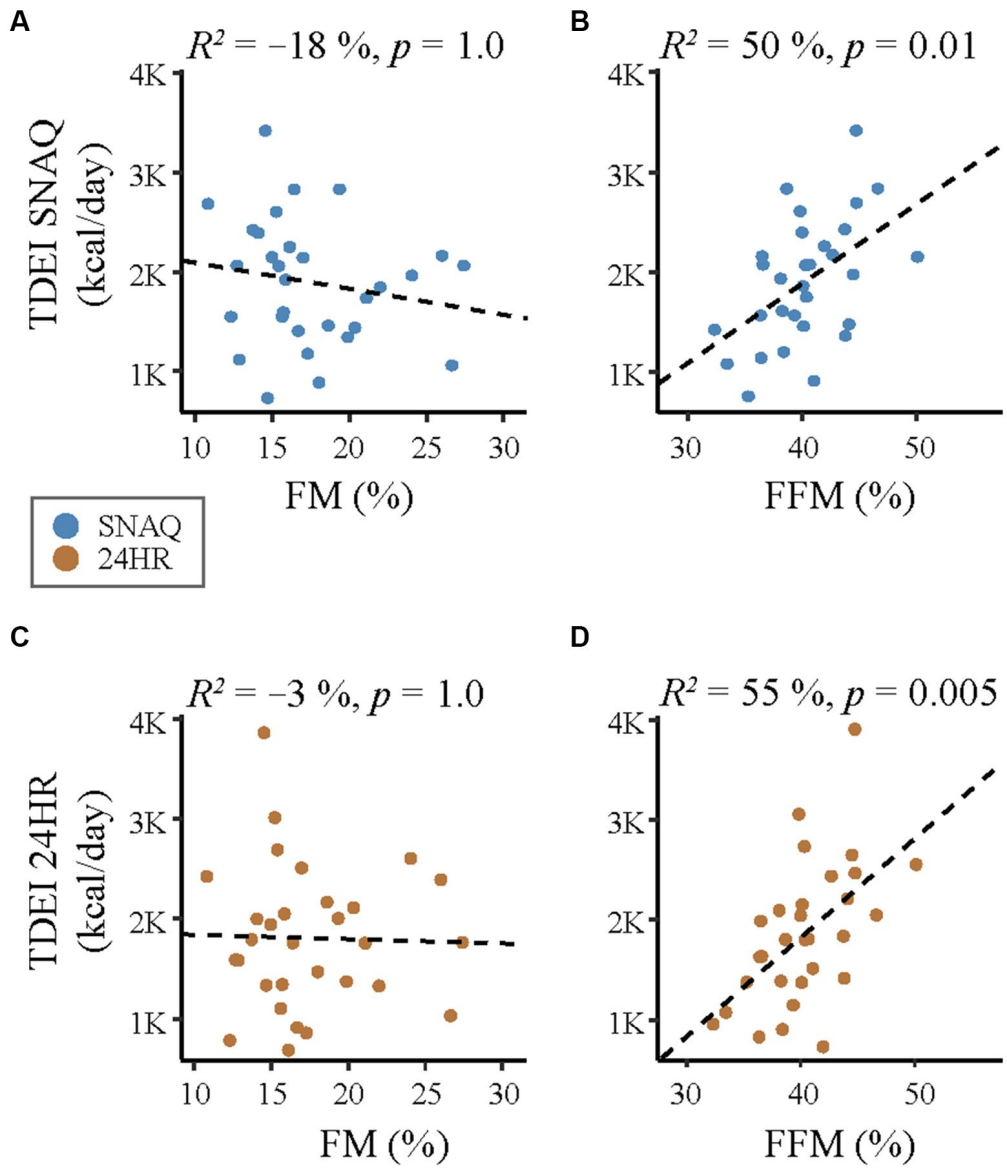
Correlation analysis of linear relationship between body composition (fat mass, FM and fat-free mass, FFM) and the energy intake estimated with the SNAQ app and the 24-hour dietary recall (24HR). **(A)** Linear correlation between daily energy intake estimated with SNAQ and FM. **(B)** Linear correlation between daily energy intake estimated with SNAQ and FFM. **(C)** Linear correlation between daily energy intake estimated with 24HR SNAQ and FM. **(D)** Linear correlation between daily energy intake estimated with 24HR and FFM. 24HR, 24-hour dietary recall; *p*, *p*-value of the coefficient of determination; *R*^2^, coefficient of determination of the linear relationship.

**Figure 7:**
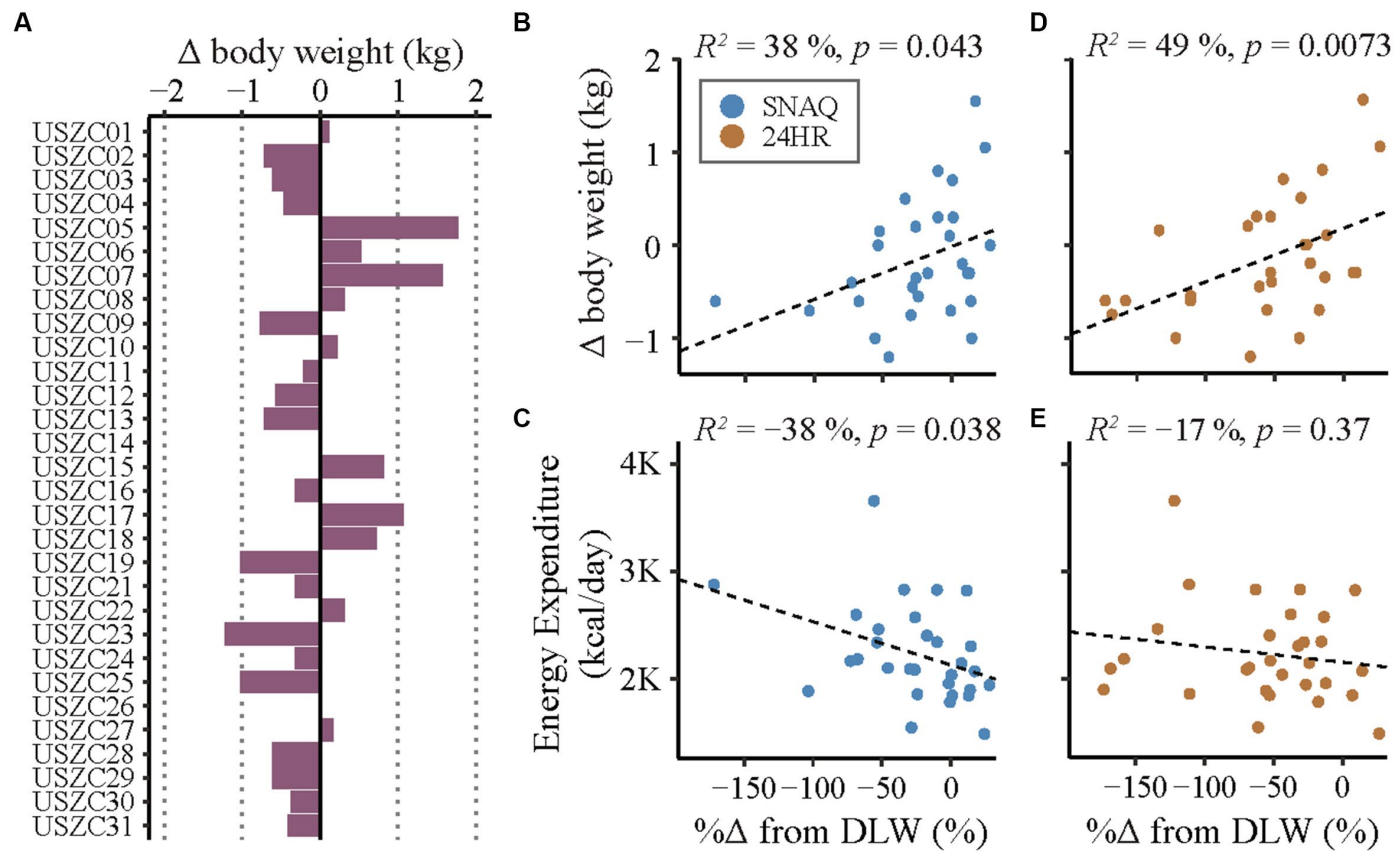
Changes in body weight during the study period and correlation analysis of linear relationships of measurement differences of the SNAQ app and the 24-hour dietary recall (24HR). **(A)** Changes of body weight of the study participants during the study week. Bars on the left of the y-axis represent body weight loss. Bars on the right of the y-axis represent body weight gain. **(B)** Linear correlation between changes of body weight during the study week and percentage of measurement differences of daily energy intake estimated with SNAQ in relation to DLW. **(C)** Linear correlation between estimations of daily energy expenditure during the study week and percentage of measurement differences of daily energy intake estimated with SNAQ in relation to DLW. **(D)** Linear correlation between changes of body weight during the study week and percentage of measurement differences of daily energy intake estimated with 24HR in relation to DLW. **(E)** Linear correlation between estimations of daily energy expenditure during the study week and percentage of measurement differences of daily energy intake estimated with 24HR in relation to DLW. Δ, difference from baseline; %Δ, percentage difference; 24HR, 24-hour dietary recall; DLW, doubly labelled water; *K*, decimal unit suffix for thousand; *p*, *p*-value of the coefficient of determination; *R*^2^, coefficient of determination of the linear relationship.

A Bland–Altman-analysis (62) was performed to assess the level of agreement between the three methods of energy estimation. This analysis is widely used to visualize the differences between two methods of medical assessment by plotting the mean measurements of the two methods (x-axis) against the differences in measurements between the two methods (y-axis). Differences were expressed in relation to the DLW values – when DLW was part of the analysis – or 24HR values. Then, lower and upper limits of agreement (LoA) were derived from the mean difference between methods ±1.96 SD (63). Possible systematic bias was assessed visually on the Bland–Altman plots. However, any level-dependent bias identified through this visual inspection was not further addressed in the analysis.

The strength of the linear relationships stated as further outcomes was evaluated using a Pearson’s correlation coefficient. The correlation coefficient (*r*) was calculated to assess the magnitude of the relationship. The effect size was interpreted based on the magnitude of the coefficient of determination (*R*^2^), with values closer to +1 or −1 indicating a stronger relationship. The *R*^2^ represents the proportion of variance in one variable that can be explained by the other variable in a linear regression model. The significance of the relationship was assessed using the corresponding *p*-value, with *p* < 0.05 considered statistically significant. The significance value of all coefficients of determination was set at 0.05, but the *p*-values were adjusted with a 3x Bonferroni correction to account for all correlation analyses performed between any variable and both SNAQ and 24HR.

The differences in energy estimates between the methods were calculated by subtracting the energy estimates of the reference method (either DLW or 24HR) from the values of TDEI estimated with SNAQ or 24HR. The percentage differences of energy estimates between the methods were calculated as percentage change from the values estimated by the DLW. A paired difference test was performed to test the null hypothesis (*H_0_*) of no difference between TDEI estimated either with SNAQ or with 24HR and TDEE estimated with DLW. The *H_0_* of no difference between TDEI estimated with SNAQ and TDEI estimated with 24HR was also tested. A *t*-test was used when the differences followed a normal distribution, while the Wilcoxon signed-rank test was used for non-normally distributed differences. The data distribution was assessed for normality using the Shapiro–Wilk test. The alternative hypothesis (*H_1_*) was a difference between TDEI estimated either with SNAQ or with 24HR and TDEE estimated with DLW, or a difference between TDEI estimated with SNAQ and TDEI estimated with 24HR. The significance value was set at 0.05, but the *p*-values were adjusted with a 3x Bonferroni correction to account for the three comparisons.

The extent of stability of diet consumption over the study week was assessed with an intraclass correlation coefficient (ICC). This provides an indication of the consistency of measurements when the measurement process is repeated under identical conditions. The ICC was calculated with a mixed-effects model as the ratio of the between-group variance to the total variance (sum of the between-group variance and the residual variance). This model considered both the variation within individuals (within-subject variation) and the variation between individuals (between-subject variation). Considering that there were 30 study participants with seven consecutive daily measurements and no intervention, a random-intercept model was fit with each study participant as a random effect. The ICC was derived from the ratio of the between-subject variance to the total variance (the sum of the between-subject variance and the within-subject variance). The ICC ranges between 0 and 1, with higher values indicating greater similarity between the day-to-day intake measurements within individuals.

The residual mean square, the between-group mean square, and the within-subject standard deviation calculated with the mixed-effects model were also reported to provide an order of effects of the model underlying the ICC. The residual mean square represents the variability of daily energy intake measurements within the same study participant. The between-group mean square represents the variability between study participants and, in the context of the ICC, this value should be much larger than the residual mean square. Indeed, a larger variability between study participants than within study participants would contribute to a higher ICC value. The within-subject standard deviation is a measure of how spread out individual scores are from the mean within the same study participants across different measurements. If this value is small relative to the between-subject variability, the ICC will be higher.

## 3. Results

### 3.1. Characteristics of the study participants

A total of 41 adult women with normal weight were invited to participate in the study. Thirty candidates were eligible according to the inclusion and exclusion criteria. The baseline characteristics of the study participants are reported in Table 1. The mean age of the participants was 28.5 (SD ± 6.7) years, and the mean BMI was 21.6 (SD ± 1.6) kg/m^2^. Among the 30 study participants, three (10%) were identified as smokers. Approximately 40% of participants had dietary preferences on which their individual diets were based. Most of the participants (87%) engaged in physical activity for at least 1–2 h per week. Body composition estimated with bioimpedance analysis and isotope dilution spaces is reported in Supplementary Table S5. Nationalities of study participants are reported in Supplementary Table S6.

**Table 1:** Baseline characteristics of the study participants.

Variable	Values *N* = 30^a^
Biological sex	
Female	30 (100%)
Age (years)	28.5 (6.7)
Height (m)	1.63 (0.07)
Weight (kg)	57.8 (6.1)
BMI (kg/m^2^)	21.6 (1.6)
Smoking^b^	3 / 30 (10%)
Dietary preferences	
None	19 / 30 (63%)
Fishetarian diet	1 / 30 (3.3%)
Lactose-free diet	3 / 30 (10%)
Vegan diet	2 / 30 (6.7%)
Vegetarian diet	5 / 30 (17%)
Physical activity (hrs/wk)	
None	5 / 30 (17%)
1–2 h/wk	5 / 30 (17%)
2–6 h/wk	16 / 30 (53%)
> 6 h/week	4 / 30 (13%)
Basal metabolic rate (kcal)	1, 383.7 (104.1)

a Mean (SD) or frequency (%). SD, standard deviation; % percentage.

b Independent of the amount of cigarettes smoked and smoking occasions.

### 3.2. Descriptive statistics and normality of energy estimates

Mean TDEI estimated with SNAQ and 24HR and mean TDEE estimated with DLW are presented with SDs in Table 2. Mean TDEI estimated with SNAQ was 1,905.5 kcal/day (SD ± 531.1), and with 24HR 1,692.1 kcal/day (SD ± 632.5). TDEE estimated with DLW was 2,235.2 kcal/day (SD ± 456.5). The results of the DLW analysis are reported in Supplementary Table S7.

**Table 2:** Energy estimates of the two dietary assessment tools and the DLW technique.

Variable	Methods of energy estimation		
	SNAQ, *N* = 30^a^	DLW, *N* = 30^a^	24HR, *N* = 30^a^
Energy intake^b^			
Energy (kcal/day)	1,905.5 (531.1)	2,235. 2 (456.5)	1,692.1 (632.5)
Energy (kJ/day)	7,972.7 (2,222.3)	9,351.9 (1,910.14)	7,079.9 (2,646.3)

Total daily energy intake was estimated with the app SNAQ and the 24-hour dietary recall (24HR). Total daily energy expenditure was estimated with the doubly labelled water (DLW) technique, but for simplicity, according to the assumption of body energy balance, in this table is referred to as energy intake.

a Mean (SD). SD, standard deviation.

b Total daily energy intake.

The values of energy were normally distributed for TDEI estimated with SNAQ and 24HR, but not for TDEE estimated with DLW (Supplementary Table S8). A visual analysis of the Quantile-Quantile (QQ) plots as well showed a larger departure of the DLW data points from their assumed normal distribution (Supplementary Figure S1A). Furthermore, an outlier was visible in QQ plots of 24HR and DLW far above the line of expected normal distribution. Nevertheless, a density plot showed that the density areas of SNAQ and DLW overlapped more than the density areas of DLW and 24HR (Supplementary Figure S1B).

### 3.3. Agreement between TDEI and TDEE

Results from the Bland–Altman analysis for agreement of TDEI estimated with SNAQ and TDEE estimated with DLW (primary outcome) and for agreement of TDEI estimated with 24HR and TDEE estimated with DLW (secondary outcome for direct comparison with an established dietary assessment tool) are shown in Table 3 and Figure 3. Bias of agreement between energy estimates from SNAQ and DLW was −329.6 kcal/day with LoA from −1,503.8 kcal/day (lower) to 844.5 kcal/day (upper) (Figure 3A). The bias of agreement between energy estimates from 24HR and DLW was −543.0 kcal/day with LoA from −1,802.5 kcal/day (lower) to 716.5 kcal/day (upper) (Figure 3B). A visual analysis of the Bland–Altman plots was negative for systemic bias at low or high values of energy.

**Table 3:** Results of the Bland–Altman plot for agreement between SNAQ and DLW, and 24HR and DLW.

Agreement between	Bias	lCI bias	uCI bias	SD bias	SE bias	lLoA	uLoA	SE LoA	lCI lLoA	uCI lLoA	lCI uLoA	uCI uLoA
SNAQ and DLW	−329.6	−553.3	−105.9	599.1	109.4	−1,503.8	844.5	189.4	−1,891.2	−1,116.3	457.0	1,231.9
24HR and DLW	−543.0	−783.0	−303.1	642.6	117.3	−1,802.5	716.5	203.2	−2218,.1	−1,386.9	300.9	1,132.1

Bias, bias of agreement; lCI: lower 95% confidence interval; lLoA, lower limit of agreement; SD, standard deviation; SE, standard error; uCI, upper 95% confidence interval; uLoA, upper limit of agreement. DLW was selected as reference method for the analysis.

### 3.4. Agreement between SNAQ and 24HR

Results from the Bland–Altman analysis for agreement between TDEI and macronutrient intake estimated with SNAQ and with 24HR (secondary outcome) are shown in Table 4 and Figure 4 (Supplementary Figure S2 for sugars and saturated fats). Bias of agreement between TDEI estimated with SNAQ and with 24HR was 213.4 kcal/day with LoA from lower −774.3 kcal/day to upper 1,201.1 kcal/day (Figure 4A). The bias of agreement was also assessed for the macronutrient intake (Figure 4B) and eating occasions (Figure 4C). Carbohydrate intake had a bias of 33.8 g/day (LoA from −101.5 to 163.7 g/day), sugar intake had a bias of 7.0 g/day (LoA from −46.0 to 60.0 g/day), fat intake had a bias of 3.4 g/day (LoA from −58.4 to 65.2 g/day), and protein intake had a bias of 2.6 g/day (LoA from −28.0 to 29.1 g/day). A visual analysis of the Bland–Altman plots was negative for systemic bias at low or high values of energy and macronutrient intake. However, a systemic bias could be identified in the Bland–Altman plot for number of eating occasions.

**Table 4:** Results of the Bland–Altman plot for agreement between SNAQ and 24HR. 24HR was selected as reference method in the analysis.

Agreement for	Bias	lCI bias	uCI bias	SD bias	SE bias	lLoA	uLoA	SE LoA	lCI lLoA	uCI lLoA	lCI uLoA	uCI uLoA
Energy^a^ (kcal)	213.4	25.2	401.6	503.9	92.0	−774.3	1,201.1	159.4	−1,100.2	−448.4	875.1	1,527.0
Energy^a^ (kJ)	892.8	105.5	1,680.1	2,108.4	384.9	−3,239.6	5,025.2	666.7	−4,603.2	−1,876.0	3,661.6	6,388.8
Carbohydrates (g)	33.8	7.7	59.9	69.8	12.7	−103.0	170.6	22.1	−148.1	−57.9	125.4	215.7
Sugars (g)	7.0	−3.1	17.1	27.0	4.9	−46.0	60.0	8.6	−63.5	−28.5	42.5	77.5
Fats (g)	3.4	−8.4	15.2	31.5	5.8	−58.4	65.2	10.0	−78.8	−38.0	44.8	85.6
Saturated fats (g)	−2.1	−8.7	4.5	17.7	3.2	−36.9	32.7	5.6	−48.4	−25.4	21.2	44.1
Proteins (g)	2.6	−8.3	13.6	29.4	5.4	−54.9	60.2	9.3	−73.9	−35.9	41.2	79.2
Fibers (g)	0.5	−4.9	6.0	14.6	2.7	−28.0	29.1	4.6	−37.4	−18.6	19.6	38.5
Eating occasions (*n*)	2.9	2.1	3.7	2.2	0.4	−1.4	7.2	0.7	−2.9	0.0	5.8	8.6

Bias, bias of agreement; lCI: lower 95% confidence interval; lLoA, lower limit of agreement; SD, standard deviation; SE, standard error; uCI, upper 95% confidence interval; uLoA, upper limit of agreement.

a Total daily energy intake.

### 3.5. Further outcomes

#### 3.5.1. Relationship between TDEI and TDEE

A correlation analysis between the TDEE estimated with DLW and the TDEI estimated with SNAQ and 24HR is shown in Figures 3C,D, respectively. There was no significant linear relationship between TDEI estimated with SNAQ and TDEE (*R*^2^ = 27%, *p* = 0.5), nor between TDEI estimated with 24HR and TDEE (*R*^2^ = 34%, *p* = 0.2).

#### 3.5.2. Differences between energy assessment tools

Absolute and percentage differences between the methods (SNAQ and DLW, and 24HR and DLW) are shown with SDs in Table 5. Individual values of all study participants are shown in Supplementary Tables S9 (for SNAQ) and S10 (for 24HR) to present the variability of the differences within this study population. The high variability in the differences between the methods is also visible in Supplementary Figure S3.

**Table 5:** Overall difference, and over- and underestimations of total daily energy intake estimated with SNAQ and 24HR in relation to DLW.

Variable		*n*	Δ DLW, *N* = 30^a^	MPD DLW, *N* = 30^a^
Overall difference	Energy intake^b^ SNAQ			
	Energy (kcal/day)Energy (kJ/day)	30	−330.0 (559.0)−1,380.7 (2,338.9)	−12.8% (25.3)
	Energy intake 24HR			
	Energy (kcal/day)Energy (kJ/day)	30	−543.0 (664.6)−2,271.9 (2,780.7)	−22.9% (29.5)
Overestimations	Energy intake SNAQ			
	Energy (kcal/day)Energy (kJ/day)	4	391.5 (171.0)1,638.0 (715.5)	19.8% (10.2)
	Energy intake 24HR			
	Energy (kcal/day)Energy (kJ/day)	10	664.4 (212.0)2,779.8 (887.0)	34.7% (18.0)
Underestimations	Energy intake SNAQ			
	Energy (kcal/day)Energy (kJ/day)	26	−591.9 (464.4)−2,476 (1,943.0)	−24.5% (17.4)
		Energy intake 24HR			
	Energy (kcal/day)Energy (kJ/day)	20	−728.8 (487.7)−3,049.3 (2,040.5)	−31.8% (18.9)

24HR, 24-hour dietary recall; DLW, doubly labelled water; MPD DLW, mean percentage difference from DLW values; *n*, number of cases; Δ DLW, difference from DLW values.

a Mean (SD). SD, standard deviation.

b Total daily energy intake.

Differences of the energy estimates of SNAQ and 24HR in relation to DLW are represented as violin plots in Figure 5A. Difference in TDEI estimated with SNAQ and the TDEE estimated with DLW was not statistically significant (−330.0 kcal/day, SD ± 559.0, *p* = 0.067). However, the *p*-value estimated for this difference was significant before adjustment with the Bonferroni correction (*p* = 0.022). There was a statistical difference between the TDEI estimated with 24HR and the TDEE estimated with DLW (−543.0 kcal/day, SD ± 664.6, *p* = 0.00059).

#### 3.5.3. Over- and underestimations of SNAQ and 24HR in relation to DLW

Despite the finding that both the SNAQ and 24HR underestimated energy intake compared to the values of TDEI expected based on TDEE in conditions of energy balance (Figure 5B), a more detailed analysis revealed instances where energy intake was overestimated (Figure 5C). These overestimations were nonetheless less frequent than underestimations and had a smaller percentage difference from TDEE. TDEI estimated with SNAQ was 12.8% lower than TDEE estimated with DLW. However, this was the result of 4 overestimations with an average of 19.8% ± 10.2% and 26 underestimations with an average of −24.5% ± 17.4%. TDEI estimated with 24HR was 22.9% lower than TDEE estimated with DLW. However, this was the result of 10 overestimations with an average of 34.7% ± 18.0% and 20 underestimations with an average of −31.8% ± 18.9%.

For the definition of study-specific Goldberg cut-off points, an *S* factor of 26.98% was calculated based on a CV_wTDEI_ of 42.55%, and CV_tP_ of 20.24%. Lower and upper 95% confidence limits were 1.51 and 1.84, respectively. According to these Goldberg cut-off points, 6 study participants had a plausible TDEI in relation to their BMR, 20 study participants were under-reporters when using SNAQ, and 4 study participants were over-reporters (Supplementary Table S11). When using 24HR to report their food intake, 3 study participants had a plausible TDEI in relation to their BMR, 25 were under-reporters, and 2 were over-reporters (Supplementary Table S12).

When using the CV_wTDEI_ of 26% suggested by Black (35) to calculate the *S* factor (20.04%), lower and upper 95% confidence limits were 1.55 and 1.79, respectively. According to these Goldberg cut-off points, 5 study participants had a plausible TDEI in relation to their BMR, 19 study participants were under-reporters when using SNAQ, and 6 study participants were over-reporters (Supplementary Table S11). When using 24HR to report their food intake, 2 study participants had a plausible TDEI in relation to their BMR, 25 were under-reporters, and 3 were over-reporters (Supplementary Table S12).

#### 3.5.4. Relationship between body composition and energy estimates

A correlation analysis between body composition (FFM and FM) estimated with DLW and the TDEI estimated with SNAQ and 24HR is shown in Figure 6. There was a moderate relationship between FFM and TDEI estimated with SNAQ (*R*^2^ = 50%, *p* = 0.01) and between FFM and TDEI estimated with 24HR (*R*^2^ = 55%, *p* = 0.005). There was no significant relationship between FM and TDEI estimated either with SNAQ (*R*^2^ = −18%, *p* = 1.0) or with 24HR (*R*^2^ = −3%, *p* = 1.0).

#### 3.5.5. Relationship between changes in body weight and measurement differences

The changes of body weight of the study participants during the study week are shown in Figure 7A. A correlation analysis of the linear relationship between the changes in body weight and the measurement differences of TDEI estimated with SNAQ and 24HR in relation to TDEE estimated with DLW is shown in Figures 7B,D, respectively. There was a moderate linear relationship between the changes in body weight and the measurement differences of 24HR (*R*^2^ = 49%, *p* = 0.015), but not with the measurement differences of SNAQ (*R*^2^ = 38%, *p* = 0.086).

#### 3.5.6. Relationship between energy expenditure and measurement differences

A correlation analysis between the TDEE estimated with DLW and the measurement error of TDEI estimated with SNAQ and 24HR in relation to TDEE estimated with DLW is shown in Figures 7C,E, respectively. There was no significant linear relationship neither between the measurement error with SNAQ and TDEE (*R*^2^ = −38%, *p* = 0.076) nor between the measurement error with 24HR and TDEE (*R*^2^ = −17%, *p* = 0.74).

#### 3.5.7. Stability of diet consumption over the study week

The intraclass correlation coefficient (ICC) was calculated among the 30 study participants to assess the reliability of TDEI measurements for each study participant over a period of seven consecutive days (Supplementary Table S13). The ICC was estimated to be 0.274 (95% confidence interval: 0.137 to 0.460, *p* < 0.001). This indicated a moderate level of agreement in the stability of diet consumption across the study week. The F-statistic was 3.269 with degrees of freedom equal to 29 and 145 for the numerator and denominator, respectively. The residual mean square was 508,507.1 (kcal/day)^2^, the between-group mean square was 217,928.3 (kcal/day)^2^, and the within-subject standard deviation was 759.0 kcal/day.

#### 3.5.8. Relationship and differences between TDEI estimated with SNAQ and 24HR

A correlation analysis between the TDEI estimated with SNAQ and the TDEI estimated with 24HR is shown in Supplementary Figure S4. There was a significant relationship between TDEI estimated with SNAQ and TDEI estimated with 24HR (*R*^2^ = 62%, *p* = < 0.001).

TDEI and macronutrient intake estimated with SNAQ and with 24HR are shown in Table 6. Absolute and percentage differences between the two methods are also reported together with a 95% confidence interval of the absolute differences. Even though there was a high variability in the differences between the two methods, as also shown in Supplementary Figure S5 and Supplementary Table S14, there was no statistical difference between TDEI estimated with SNAQ and with 24HR (213.4 kcal/day, SD ±503.9, *p* = 0.6). TDEI reported with SNAQ and with 24HR were 1,905.5 ± 531.1 kcal/day and 1,692.1 ± 632.5 kcal/day, respectively.

**Table 6:** Comparison of energy intake, macronutrient intake, and dietary occasions between SNAQ and 24HR.

Variable	Dietary assessment method		Difference^c^	95% CI^c^	*p*-value^c^
	SNAQ, *N* = 30^a^	24HR, *N* = 30^a^			
Energy intake^b^					
Energy (kcal/day)	1,905.5 (531.1)	1,692.1 (632.5)	213 (+23%)	−89, 515	0.2
Energy (kJ/day)	7,972.7 (2,222.3)	7,079.9 (2,646.3)	893 (+23%)	−371, 2,157	0.2
Macronutrient intake					
Carbohydrates (g/day)	213.07 (68.83)	179.27 (94.80)	34 (+38%)	−9.1, 77.0	0.12
Sugars (g/day)	70.69 (27.75)	63.70 (30.16)	7.0 (+28%)	−8.0, 22.0	0.4
Fats (g/day)	76.65 (30.69)	73.27 (28.47)	3.4 (+16%)	−12.0, 19.0	0.7
Saturated fats (g/day)	25.39 (8.46)	27.50 (17.82)	−2.1 (−18%)	−9.4, 5.2	0.6
Proteins (g/day)	72.64 (22.09)	70.00 (39.22)	2.6 (+23%)	−14.0, 19.0	0.7
Fibers (g/day)	21.43 (10.62)	20.89 (14.45)	0.53 (+30%)	−6.0, 7.1	0.9
Temporal organization					
Eating occasions (*n*/day)	7.5 (1.9)	4.6 (1.3)	2.9 (+72%)	2.0, 3.7	**< 0.001**

CI, Confidence interval.

a Mean (SD). SD, standard deviation.

b Total daily energy intake.

c Welch Two Sample t-test.

Similarly, there was no statistical difference for the mean intake of any of the macronutrients estimated with SNAQ and with 24HR. Nonetheless, there was a statistical difference in the number of eating occasions reported with the two methods (D = 2.9, *p* < 0.001). Number of eating occasions reported with SNAQ and with 24HR were 7.5 ± 1.9 and 4.6 ± 1.3, respectively. Differences between the estimates of the two methods are represented as boxplots in Supplementary Figure S6.

#### 3.5.9. Over- and underestimations of SNAQ in relation to 24HR

Percentage differences of SNAQ in relation to 24HR for estimates of TDEI and macronutrient intake are reported in Table 7. Individual values of all study participants are shown in Supplementary Figure S7 to present the variability of the differences within this study population. Even though the overall percentage difference between SNAQ and 24HR indicates an overestimation of intake by SNAQ for TDEI and all considered macronutrients, except for saturated fats, a closer analysis also showed underestimations. Underestimations were however less frequent than overestimations and had a smaller percentage difference from 24HR. For instance, TDEI estimated with SNAQ was 23% higher than with 24HR. However, this was the result of 18 overestimations with an average of 49.0 ± 48.1% and 12 underestimations with an average of −15.3 ± 7.9%. Overestimations for macronutrient intake were 38% for carbohydrates, 28% for sugars, 16% for fats, 23% for proteins, and 30% for fibers.

**Table 7:** Over- and underestimations of energy intake estimated with SNAQ in relation to 24HR.

Variable		*n*	Δ 24HR, *N* = 30^a^	Δ% 24HR, *N* = 30^a^
Overestimations	Energy intake^b^			
	Energy (kcal/day)	*18*	559.0 (359.0)	48.8% (48.1)
	Energy (kJ/day)	*18*	2,338.8 (1,502.1)	48.8% (48.1)
	Macronutrient intake			
	Carbohydrates (g/day)	*19*	77.4 (41.8)	69.6% (56.6)
	Sugars (g/day)	*18*	24.8 (17.7)	64.7% (61.6)
	Fats (g/day)	*17*	24.5 (24.1)	50.4% (68.6)
	Saturated Fats (g/day)	*17*	8.0 (7.4)	59.8% (81.0)
	Proteins (g/day)	*18*	24.2 (11.0)	55.7% (36.7)
	Fibers (g/day)	*20*	7.1 (5.1)	69.6% (69.1)
	Temporal organization			
	Dietary occasions (*n*/day)	*28*	3.2 (1.8)	79.2% (51.2)
Underestimations	Energy intake			
	Energy (kcal/day)	*12*	−305.0 (128.3)	−15.3% (7.9)
	Energy (kJ/day)	*12*	−1,276.2 (536.7)	−15.3% (7.9)
	Macronutrient intake			
	Carbohydrates (g/day)	*11*	−41.6 (35.1)	−15.6% (10.6)
	Sugars (g/day)	*12*	−16.3 (14.2)	−20.4% (16.1)
	Fats (g/day)	*13*	−20.7 (19.0)	−23.8% (18.2)
	Saturated Fats (g/day)	*13*	−15.3 (17.3)	−35.7% (18.8)
	Proteins (g/day)	*12*	−29.7 (30.4)	−25.5% (17.5)
	Fibers (g/day)	*10*	−9.4 (9.9)	−27.4% (24.6)
	Temporal organization			
	Dietary occasions (*n*/day)	*2*	−1.9 (0.6)	−26.6% (8.7)

Δ 24HR, difference from 24HR values; Δ% 24HR, percentage difference from 24HR values; *n*, number of over- or underestimations per variable.

a Mean (SD). SD, standard deviation.

b Total daily energy intake.

## 4. Discussion

This study aimed to assess the validity of an image-based food-recognition app, SNAQ, for dietary assessment of daily energy intake, in free-living conditions, of adult women with normal body weight. The validity of the app was assessed in terms of agreement with a reference method for energy expenditure, the DLW technique. Further, the agreement of SNAQ with 24HR for dietary assessment of energy and macronutrient intake was also assessed.

In general, energy intake estimated with SNAQ provided a closer representation of the energy expenditure estimated with DLW than the energy intake estimated with 24HR, while there were no significant differences between energy and macronutrient intake estimated with SNAQ and 24HR.

The comparison of our findings with previous studies is difficult since there are significant variations in the way others have previously evaluated the accuracy of dietary assessment tools. Such variations can be attributed, in part, to the statistical methods employed to determine how well a given dietary assessment tool agrees with a reference method. To address this issue, we conducted a comprehensive assessment utilizing four statistical and three descriptive approaches to assess differences and relationships between energy intake estimated with SNAQ and 24HR and the energy expenditure estimated with DLW. Our objective was to measure agreement at both the individual and group levels, with the aim of providing reliable evidence for the validity of SNAQ as a dietary assessment tool.

To begin with, the Bland–Altman analysis showed that energy intake estimated with SNAQ had a bias of −329.6 kcal/day in relation to the energy expenditure estimated by DLW. This bias was 213.4 kcal/day smaller than the one estimated with the established tool, 24HR. Therefore, both dietary assessment tools seemed to have a bias in the same direction and very large LoA for energy estimates. Additionally, a correlation analysis showed no significant linear relationship between the energy estimates of both dietary assessment tools with the energy expenditure estimated with DLW.

Furthermore, the difference between the energy intake estimated with SNAQ and the energy expenditure estimated with DLW was significantly different, but the difference lost significance when the *p*-value was adjusted with the Bonferroni correction. Therefore, it is not clear in the present study population if the difference was due to chance alone or not. Indeed, there is an ambiguity of equivalence between DLW and SNAQ energy estimates at the population level which cannot be investigated with an equivalence test due the absence of a pre-established acceptable equivalence value for boundary setting. However, there was a strong significant difference between energy intake estimated with 24HR and energy expenditure estimated with DLW. Indeed, it has also previously been reported that the 24HR underestimates energy intake when compared to reference methods, such as the DLW technique (7). Based on our findings, it appears that SNAQ exhibits greater overall validity in assessing energy intake in our study population in comparison to 24HR.

The results of our study were partly consistent with previous research that compared other *image-based* apps for dietary assessment to DLW (10, 11, 43). Martin et al. (*n* = 13) and Most et al. (*n* = 23) reported a bias of −270 kcal/day and − 600 kcal/day, respectively, but both did not find a significant difference between the methods (10, 43). Gemming et al. reported a bias of −180 kcal/day compared to a bias of −340 kcal/day of a traditional tool (11) but also a significant difference for estimations of energy intake between the investigated image-based dietary tool and DLW at the group level. Boushey et al. (*n* = 30) reported a bias of −563 kcal/day and also found a significant difference between methods, but reported a strong linear relationship between the methods (64).

Based on the combination of the results of the Bland–Altman plot, of the paired difference tests and of the Parsons’s correlation coefficient, these authors provided different conclusions. Martin et al. and Gemming et al. both concluded that the dietary assessment tool under investigation showed good relative validity for the target population, despite Gemming found a significant difference from the reference method. Instead, Most et al., with no significant difference between methods but a larger bias, concluded that the image-based app that they investigated did not accurately measure energy intake when compared to DLW. Nevertheless, in these three studies, all authors all recommended caution when using the app for assessing individual energy intake. The determination of an acceptable agreement, however, remains ultimately a clinical decision, as it involves considering the context and purpose of the measurement. While statistics provide quantitative information, they cannot solely answer the question of what constitutes an acceptable clinical validity (65). In contrast to this assessment approach of validity, Boushey et al. declared a “superior position” of the investigated image-based dietary assessment tool in comparison with other assessment tools even when reporting a significant difference from the reference method (DLW) and the limits of agreement (LoA) were wider than the ones we report (64). They supported their conclusion with the significant strength of a linear relationship assessed with a Pearson’s correlation coefficient (*R^2^* = 58%, *p* < 0.0001) and an average underreporting of their women population of 16%. However, although the two methods may have been linearly related to the same direction, the fact that the two methods were significantly different when tested with a paired difference test suggests that they differed systematically.

The over- and underestimations of energy intake were assessed also in our study population. Interestingly, on the group level, the underestimation of daily energy intake with SNAQ in relation to DLW was −12.8%. However, this observation on the overall study population was led by the compensation of under- and overestimations at the individual level. While the former – the underestimation at the group level – might be relevant in the comparison of different target populations, the latter – the observation of individual differences – is of relevance when testing the validity of a method for clinical use. Indeed, any conclusion on clinical validity must consider measurement differences at the individual level. Accordingly, we performed an analysis of individual misreporting using Goldberg cut-off points specific of this study population. Most of the study participants were classified as under-reporters and some as over-reporters. In our study population, only 6 study participants had a plausible energy intake in relation to their basal metabolic rate.

Energy intake constitutes the fundamental basis of any given habitual diet. The observed underestimation of energy intake, a common occurrence in various dietary assessment tools including the SNAQ, can have implications on the assessment of macro- and micronutrient intake. Therefore, if energy intake is underestimated, it can be assumed that the intake of macro- and micronutrients is also underestimated. This limitation might restrict the broader application of SNAQ in addressing research questions beyond energy intake.

Our study represents the first attempt to assess the validity of SNAQ for assessment of energy intake by comparing the estimated energy intake to a reference method for energy expenditure. It is difficult to define if the limits of agreement that we report with the Bland–Altman plot can be considered acceptable for clinical use as component of diagnostics or in nutritional counselling. Indeed, a consensus in this regard has, to our knowledge, never been published (33). Nevertheless, the lower bias of SNAQ compared to 24HR and DLW, and the absence of a significant difference between energy estimates of SNAQ and DLW suggests that such a dietary assessment tool can have an application in research. However, based on the design of our study, it is not possible to distinguish whether the observed underestimation can be attributed to participants’ self-reports in the real-life setting or to the shortcomings of the SNAQ technology in accurately assessing the energy content of the reported food intake. This reflects a limitation and further investigations are required to validate the use of SNAQ for clinical and research purposes.

The application of SNAQ for the assessment of dietary habits assumes that the period of recording is representative for the habitual diet of the study population. This, however, might have not been the case for our study population. The intraclass correlation coefficient (ICC) of the study population for daily energy intake was moderate (0.274). This is an index of the within-subject variability during the week of recording. However, there is no universally defined acceptable value for the ICC of daily energy intake in adult women with normal body weight, as it can vary depending on the specific context and research question. Furthermore, this coefficient is generally not reported in other validity studies for new dietary assessment tools. Therefore, also a comparison of this coefficient with other similar studies is not possible. Nevertheless, this result together with the individual Goldberg classification of misreporting, suggested that some days of the study period might have been more representative than others for our study population. However, this hypothesis cannot be tested with a two-point protocol of the DLW technique. An eventual application of SNAQ for the assessment of energy intake in a clinical setting should consider the results of this study with caution and a further investigation with a multiple-point protocol of the DLW technique should be performed.

The dilution space ratio of DLW in the TBW of the study participants was in a range of 0.992–1.036 (Supplementary Table S15), with only one value below the significant threshold of 1.000 and no value above the significant threshold of 1.700. Similarly, the elimination rate ratio of DLW was in a range of 1.193–1.366 (Supplementary Table S16), with no value outside the acceptable range of 1.100–1.700. Therefore, estimations of energy expenditure with DLW were considered reliable for this study. Any measurement error between SNAQ and DLW was considered to be led by wrongful estimations of energy intake with SNAQ. However, despite providing a clear protocol to study participants regarding activities that could potentially deplete isotopes, such as limiting sports activities and sauna usage, we cannot guarantee that these activities were completely restricted, as the energy expenditure was assessed in free-living conditions.

The variability of the measurement differences with SNAQ – intended as energy intake estimated with SNAQ minus the energy expenditure estimated with DLW – could not be explained neither with changes in body weight during the study week (Figures 7B,D) nor with the estimated energy expenditure (Figures 7C,E). However, there was a significant linear relationship between changes in body weight during the study week and measurement differences of 24HR. This might suggest that with a 24HR study participants report less dietary intake than what they daily consume in the status of energy balance.

According to the assumption of energy balance, the energy intake during the study week should have equaled the energy expenditure (23). Any positive or negative difference between energy intake and energy expenditure should have resulted in an increase or decrease of the body’s energy stores, respectively. Consequently, if the changes in body weight could not explain the variability of the measurement differences with SNAQ, it might be that there might have been other reasons for these measurement differences. Nevertheless, body composition, in terms of FFM and FM, would have been a better indicator of the energy stores of the body instead of the sole body weight. Indeed, most of the energy of the body is stored in the FM (9.5 kcal/g of FM) (66–69). On one hand, the body weight also accounts for fluctuations of TBW, which contains virtually no energy. On the other hand, if the energy imbalance is the underlying cause of the measurement differences, changes in FM and FFM can be utilized to accurately determine the energy imbalance, resulting in an equal value to the individual measurement difference (68, 70).

This last approach, called the energy expenditure/balance (EB) method, involves integrating measurements of energy expenditure and changes in energy stores to estimate energy intake (22). The changes in body’s energy stores (ΔES) can be calculated by assessing the changes in FFM and FM between measurements (71). This is achieved by multiplying the changes in FFM and FM by their respective energy density coefficients (9.5 kcal/g for FM and 1.1 kcal/g for FFM). The resulting values are then divided by the number of days between the measurements to obtain the daily energy change in kcal/day.

Body composition is an important factor of energy balance. In this study, body composition was assessed with both bioelectrical impedance and isotope dilution. The bias of agreement of the two methods for TBW, FFM, and FM was comparable to what is reported on the literature (Supplementary Table S17) (72, 73). Therefore, TBW assessed using bioimpedance analysis could potentially serve as a cost-effective substitute of isotope dilution for measuring the energy stores of the body, with an acceptable decrease in precision.

Our findings regarding the secondary outcome are consistent with previous research that compared other *image-based* apps for dietary assessment to 24HR (74, 75). These studies showed no significant difference for estimations of energy intake between the apps and 24HR. The limits of agreement ranged from −549 kcal to 836 kcal and from −807 kcal to 775 kcal, respectively. In both studies, the authors concluded that the image-based app under investigation showed good relative validity when compared to 24HR for assessing energy intake at the population level. However, they recommended caution when using the app for assessing individual energy intake.

Comparable results have also been reported regarding the estimation of macronutrient intake when comparing image-based apps to 24HR (74, 75). Although there were no significant differences in macronutrient intake estimations between the methods, LoA for all macronutrients were found to be wide. These findings were confirmed in 2020 in a meta-analysis (76). It is worth noting that in all studies reviewed in the meta-analysis, the energy and macronutrient intake from images recorded in the apps was estimated by trained dietitians, whereas in our present study, we utilized the artificial intelligence feature of the app.

Further, we observed that the app tended to overestimate energy intake and all macronutrients considered, except for saturated fats, in comparison to a well-established dietary assessment tool, namely the 24HR. This does not necessarily indicate a lower validity of the app compared to the 24HR. Indeed, it has previously been reported that, when compared to reference methods, such as the DLW technique, the 24HR underestimates energy intake (7). Accordingly, it is possible that the overall validity of the app would be higher than that of 24HR when compared to the DLW technique. Indeed, as reported in the discussion, the energy intake estimated with SNAQ was not significantly different from the energy expenditure estimated with DLW, while the energy intake estimated with 24HR was significantly different from the energy expenditure estimated with DLW.

Other reasons for the increase of intrinsic measurement differences might be (1) a low accuracy of the technology in recognizing the food items or in estimating their portions, and (2) the increase of events of non-compliance of study participants (20). An earlier study previously investigated the use of SNAQ for estimation of energy and macronutrient intake. However, the research question of the mentioned study focused solely on accuracy of estimations in a laboratory setting. Our study expands on this previous work by assessing the validity of SNAQ for the estimation of energy and macronutrient intake in a real-life setting. Although highly accurate volume estimation, high segmentation performance, and low processing time were previously reported (20), in a real-life setting it is important to consider the potential influence of a greater variety of food items and eating occasions, as well as the usability of the app by non-trained users. These could affect the validity of the app in real-life scenarios.

Events of non-compliance, defined by other authors also as misreporting (77–79), are here intended as either non-recording of a dietary item or wrongful entry of food items and their portions. However, explanation on possible sources of measurement differences was out of the scope of this study. The semi-customized prompts were set within the app to overcome a potential measurement error of energy intake from non-recording due forgetfulness. This strategy has previously been reported by other authors to decrease the bias of agreement between an image-based app and DLW (43). However, due to the individual variation of snacking times, study participants did not receive notifications to record snacks.

Furthermore, the awareness of participating in a clinical study might have caused for the study participants intentional and unintentional changes of dietary behavior during the study period. Consequently, these changes might have led to an energy imbalance and have been detected as measurement differences of SNAQ. These differences are widely discussed when applying traditional dietary assessment tools such as food records and 24HR (80). However, our results did not support this possibility, because the hypothesis of energy imbalance, as already explained, could not be tested with the body weight alone.

### 4.1. Strengths

A notable strength of this study was the comparison of the energy intake estimated with SNAQ to the energy expenditure estimated with the DLW technique. This technique is considered a reference method for measures of total energy expenditure in conditions of energy balance without interfering with the routine lives of the study participants. Furthermore, the isotope dilution of the DLW was used in this study to estimate the body composition (TBW, FFM, and FM), and physical activity level (PAL) of the study participants (81, 82). Given the high cost of DLW and the considerable methodological and analytical requirements associated with it, many researchers face limitations in using this method. As a result, our study offers a valuable contribution to the field of dietary assessment by assessing the validity of a new image-based dietary assessment tool in relation to established biomarkers of energy expenditure.

Our study is the first to investigate *image-based* dietary assessment by artificial intelligence through computer vision. Most published studies using apps with artificial intelligence features are based on *image-assisted* dietary assessment methods (17, 21). So far, there has been only one published study that evaluated the validity of *image-based* food recognition through artificial intelligence in free-living conditions (21). Furthermore, one significant advantage of using the SNAQ app was its integration with the Swiss Food Composition Database (57), which might have facilitated study participants in recalling their dietary intake with comprehensive database specific to the local cuisine.

Our study benefits from the recruitment of a homogeneous study population of adult women with normal body weight. By employing this approach, the potential influence of confounding variables that might affect the outcomes is effectively minimized. This enhances the internal validity of the study, ultimately increasing the reliability of the collected data. However, it is possible that this approach limits the external validity of the findings, constraining the applicability of the findings to other source populations. To enhance the overall generalizability of the results, future research works should consider larger sample sizes and broader inclusion criteria, such as obesity, to obtain more representative results.

### 4.2. Limitations

A noteworthy limitation of the research design employed in these studies is the absence of measures to monitor compliance among participants with regard to the meal recordings. For instance, adequate lighting, type of plate used to place the food items, and distance of the smartphone to the meals was not supervised, as the participants were in a free-living condition. Moreover, the evaluation of the feasibility, user-friendliness and participant satisfaction with SNAQ was not carried out, which could potentially lead to inaccurate monitoring of dietary intake owing to the app’s laborious recording process.

The use of the DLW technique as a reference method for energy expenditure is based on the assumption of energy balance during the study period. However, a seven-day protocol might have been too short to capture significant changes in the metabolism of the study participants due to adaptation to the energy intake and energy expenditure influenced by the participation in the study. A period of 14 days might still be a reasonable solution between feasibility of the study and quality of data (22). Indeed, the accuracy of the DLW technique is influenced by the dosage of labeled water administered and the duration of the metabolic period. In adults, the optimal metabolic period, which corresponds to 1.5 half-lives of the tracers in the body, typically ranges from 10 to 14 days (22). This duration is critical for achieving precise measurements with DLW.

The use of 24HR as a method for comparison also poses several limitations. The self-reported nature makes this method prone to underreporting (1). Furthermore, intake data from 24HR was only collected for 1 day, whereas data collected from the app represented the average intake over 7 days. It is therefore possible, that the 24HR depicted an unusual day, regarding eating behavior (particularly high or low intake). Conducting multiple 24HRs for the same participant and on week-, as well as weekend days could provide more accurate information on the eating behavior of the participants and reduce random error. However, there is confusion in the literature whether one or the mean of several 24HR should be used, as systematic bias of the method might remain (83).

Physical activity during the study period was not measured. However, the general assumption that higher levels of physical activity result in increased TDEE (24, 25), leading to a negative energy balance and subsequent weight loss, has been rejected (26). Indeed, when individuals engage in high levels of physical activity, the TDEE does not continue to increase proportionally. Instead, the body undergoes adaptations to maintain energy balance. These adaptations occur through both behavioral changes, such as reducing non-exercise physical activity, and metabolic adjustments, such as changes in metabolic efficiency. As a result, the increase in TDEE is limited. The body strives to maintain energy balance rather than experiencing a linear increase in energy expenditure with higher physical activity levels. Nevertheless, monitoring of physical activity during the study period might be important to explain differences between energy expenditures and energy intake, and therefore changes in energy stores of the body. This monitoring allows for a more comprehensive assessment of energy balance and provides insights into the factors influencing energy expenditure and energy intake.

To assess the validity of a new dietary assessment tool, various statistical methods are commonly employed. These methods help determine the accuracy and reliability of the tool by comparing it to an established reference method. However, there is currently no consensus on which statistical methods should be used for assessing validity. Consequently, different approaches can be used, making it challenging to compare and interpret the results across studies.

To address this issue, our suggestion is to establish a benchmark of statistical methods that should be included in research investigating the validity of dietary assessment tools. The idea behind this benchmark is to provide a standardized set of statistical methods and descriptive calculations that researchers should report and use consistently, allowing for better comparison and interpretation of results across studies.

**Figure 8:**
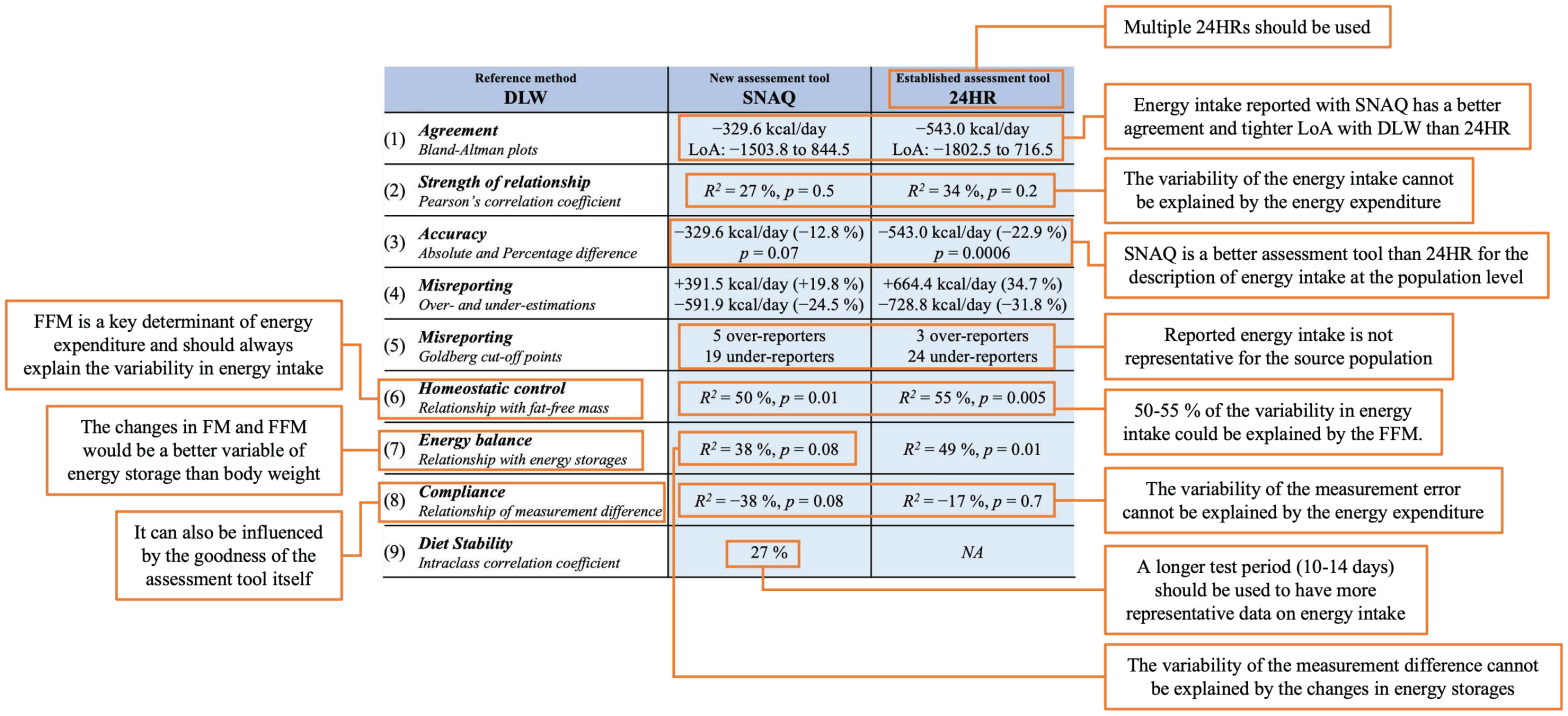
Overview of the proposed benchmark for standardized reporting of nine specific statistical and descriptive steps for assessment of validity of a new dietary assessment tool. The different statistical methods and descriptive calculations are illustrated in the table in the middle of the figure. The interpretation of the single values generated by each method and calculation is explained in the orange-margined text boxes.

In our proposal, we recommend including nine specific statistical and descriptive steps in the benchmark for assessing validity: (1) Bland–Altman plots for assessment of agreement, (2) Pearson’s correlation coefficient of the strength of relationship between methods, (3) absolute and percentage measurement differences between the methods for assessment of relative accuracy, (4) over- and underestimations of the new dietary assessment tool compared to the reference method for assessment of misreporting, (5) calculation of Goldberg cut-off points for the investigated study population for identification of individual over- and under-reporters, (6) Pearson’s correlation coefficient of the strength of relationship between body composition (FM and FFM) and the energy intake estimated with the new dietary assessment tool for assessment of the homeostatic control, (7) Pearson’s correlation coefficient of the strength of relationship between changes of energy stores of the body and the measurement differences between the methods for assessment of energy balance, (8) Pearson’s correlation coefficient of the strength of relationship between energy expenditures and the measurement differences between the methods for assessment of compliance, and (9) intraclass correlation coefficient for assessment of diet stability. These methods likely have been identified as suitable and informative for evaluating the accuracy and reliability of dietary assessment tools. An overview of the benchmark is presented in Figure 8, illustrating the different statistical methods and descriptive calculations, and explaining how to interpret the single values generated by each method.

Lastly, the assessment of diet is a complex process that needs the utilization of precise and consistent different methods. It is important to notice that clinicians and researchers should not depend exclusively on new technologies to address all the challenges related to dietary assessment when attempting to evaluate diet. The selection of an appropriate assessment method is contingent upon various factors, such as the research question, study design, characteristics of the sample, and sample size, among others. Dietary assessment tools which directly assess food intake should be implemented in order to understand the direct connection between diet and health. Despite the technological advancements, the intrinsic individual bias that is linked to self-reported dietary intake cannot be eliminated by such novel tools employed to evaluate dietary intake. Consequently, it is essential to incorporate new techniques that enable a *direct* measurement of dietary intake into the field of nutrition research, such as residential facilities (84, 85), the Universal Eating Monitor (86) or the drinkometer device (87).

## 5. Conclusion

The selected *image-based* food-recognition app, SNAQ, provided an estimate of energy intake of the study population which was not significantly different from the energy expenditure estimated with the DLW technique. Further, there was no significant difference between SNAQ and 24HR for estimates of energy and macronutrient intake.

Despite SNAQ showing slightly better performance in relation to DLW compared to 24HR, it is not possible to conclude that SNAQ has a higher validity than 24HR for assessing energy intake in adult women with normal weight. Future research work is encouraged to further explore and address the limitations identified in this study. Energy intake is underestimated, the limits of agreement are wide, and, even if there is no significant difference between SNAQ and DLW at the group level, it must be investigated if the measurement difference between the methods is clinically relevant. Furthermore, the agreement of the app with the DLW technique remains to be assessed in other target populations which might profit by the implementation of this dietary assessment tool.

Finally, it is necessary to acknowledge that SNAQ still lacks accuracy in estimating energy intake and further technologies should be explored to assess food intake in humans.

## Data availability statement

The raw data supporting the conclusions of this article will be made available by the authors, without undue reservation.

## Ethics statement

The studies involving humans were approved by Ethics Commission of Zurich, Canton of Zurich, Switzerland. The studies were conducted in accordance with the local legislation and institutional requirements. The participants provided their written informed consent to participate in this study.

## Author contributions

MS: Data curation, Formal analysis, Investigation, Methodology, Project administration, Resources, Software, Supervision, Validation, Visualization, Writing – original draft, Writing – review & editing. DA: Conceptualization, Investigation, Writing – original draft, Writing – review & editing. FH: Data curation, Writing – original draft. PH: Conceptualization, Methodology, Writing – review & editing. HM: Formal Analysis, Methodology, Writing – review & editing. AT: Investigation, Methodology, Writing – review & editing. RS: Funding acquisition, Supervision, Writing – review & editing. PG: Investigation, Methodology, Resources, Writing – review & editing. AS: Conceptualization, Methodology, Supervision, Writing – review & editing. DG: Conceptualization, Writing – review & editing. MB: Conceptualization, Funding acquisition, Supervision, Writing – review & editing.

## Figures and Tables

**Table:** 

BIA	Bioelectrical Impedance Analysis
BMR	Basal Metabolic Rate
CV	coefficient of variation
CV_tP_	between-subject variation of Physical Activity Level
CV_wB_	coefficient of variation of repeated BMR measurements
CV_wTDEI_	mean within-subject coefficient of variation for TDEI reported with SNAQ
FFM	Fat-Free Mass
FM	Fat Mass
LoA	limit of agreement of a Bland–Altman plot
PAL	Physical Activity Level
*r*	Pearson’s correlation coefficient
*R* ^2^	coefficient of determination derived from a Pearson’s correlation coefficient
SD	1.96 Standard Deviation
SNAQ	the SNAQ study app developed by SNAQ AG (Switzerland) for the purpose of this study
TBW	Total Body Water
TDEE	Total Daily Energy Expenditure
TDEI	Total Daily Energy Intake
24HR	24-h dietary recall
