# Supplementary material for: Assessing daily energy intake in adult women: validity of a food-recognition mobile application compared to doubly labelled water
Source: Front Nutr. 2023 Sep 22;10:1255499. doi: 10.3389/fnut.2023.1255499 (PMC10556674; doi:10.3389/fnut.2023.1255499)
Supplement: Supplementary file 1 [file Data_Sheet_1.docx]

Assessing Daily Energy Intake in Adult Women: Validity of a Food-Recognition Mobile Application compared to Doubly Labelled Water

Michele Serra, M.Sc.^1,2†^, Daniela Alceste, M.Sc.^1,2†^, Florian Hauser, MD^2^, Paul J. M. Hulshof, PhD^3^, Harro A. J. Meijer, PhD^4^, Andreas Thalheimer, MD^1^, Robert E. Steinert, PhD^1^, Philipp A. Gerber, MD, PD^5^, Alan C. Spector, PhD^6^, Daniel Gero, MD, PhD^1^, Marco Bueter, MD, PhD^1^*

^1^ Department of Surgery and Transplantation, University Hospital Zurich, Zurich, Switzerland.

^2^ Faculty of Medicine, University of Zurich (UZH), Zurich, Switzerland.

^3^ Division of Human Nutrition, Wageningen University, Wageningen, The Netherlands.

^4^ Centre for Isotope Research (CIO), Energy and Sustainability Research Institute Groningen, University of Groningen, The Netherlands.

^5^ Department of Endocrinology, Diabetology and Clinical Nutrition, University Hospital Zurich, Zurich, Switzerland.

^6^ Department of Psychology and Program in Neuroscience, Florida State University, Tallahassee, FL, USA.

† These authors contributed equally to this work and share first authorship

*** Correspondence:**Prof. Dr. med. Marco Bueter, PhD

Department of Surgery and Transplantation, University Hospital Zurich, Switzerland

Email: marco.bueter@usz.ch

Phone: +41 (0)44 255 8895

Fax: +41 (0)44 255 8941

Keywords: Dietary intake_1_, energy intake_2_, dietary assessment_3_, mHealth_4,_ photographic food recognition_5_, Doubly Labelled Water_6_, artificial intelligence_7_

Supplementary Material

[Supplementary figures 3](#_Toc143510596)

[Figure S1 3](#_Toc143510597)

[Figure S2 4](#_Toc143510598)

[Figure S3 5](#_Toc143510599)

[Figure S4 6](#_Toc143510600)

[Figure S5 7](#_Toc143510601)

[Figure S6 8](#_Toc143510602)

[Figure S7 9](#_Toc143510603)

[Supplementary tables 10](#_Toc143510604)

[Table S1 10](#_Toc143510605)

[Table S2 17](#_Toc143510606)

[Table S3 18](#_Toc143510607)

[Table S4 19](#_Toc143510608)

[Table S5 20](#_Toc143510609)

[Table S6 21](#_Toc143510610)

[Table S7 22](#_Toc143510611)

[Table S8 23](#_Toc143510612)

[Table S9 24](#_Toc143510613)

[Table S10 25](#_Toc143510614)

[Table S11 26](#_Toc143510615)

[Table S12 27](#_Toc143510616)

[Table S13 28](#_Toc143510617)

[Table S14 29](#_Toc143510618)

[Table S15 30](#_Toc143510619)

[Table S16 31](#_Toc143510620)

[Table S17 32](#_Toc143510621)

# Supplementary figures

Figure S1

Analysis of normal distribution of the data. **A**, Quantile-Quantile plots of energy estimates of the SNAQ app, the 24-hour dietary recall (24HR), and the doubly labelled water (DLW) technique. The diagonal dashed line represents the perfect match between the observed (empirical) and theoretical quantiles. If the data follows the theoretical distribution, the points will closely align with this line. 95 % confidence intervals are above and below the diagonal line. **B**, Density plots of the energy estimates of the three methods.


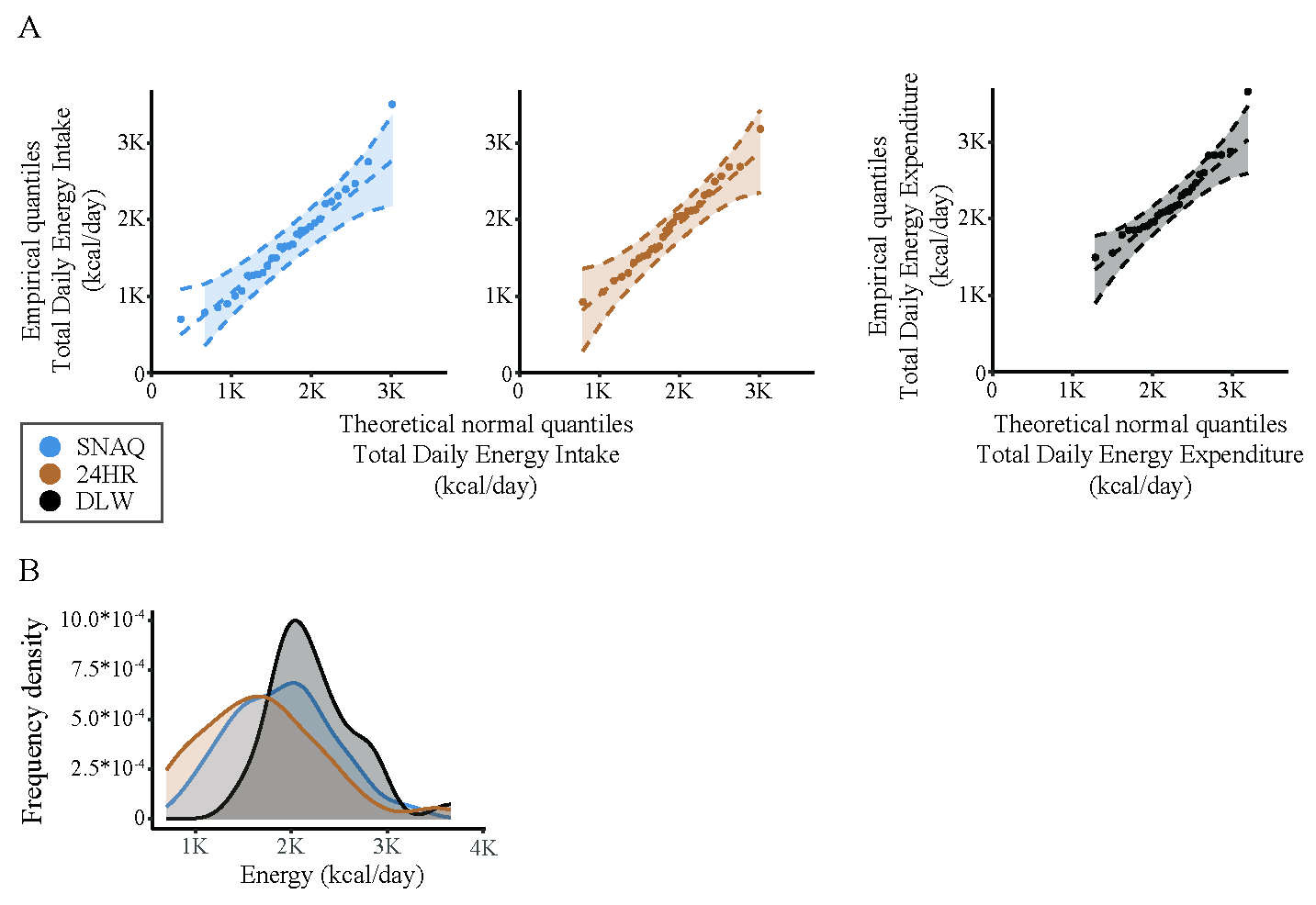


Figure S2

Bland-Altman plots for agreement between the app and the 24HR. **A**, Bland-Altman plot for sugar intake. **B**, Bland-Altman plots for intake of saturated fats. The bias is represented as a black horizontal line. The value of the bias is estimated by the mean difference in intake estimation between 24HR and the app for total daily energy intake and macronutrient intake. The 95% limits of agreement (LoA) are represented as two dotted lines and are defined as mean difference ± 1.96 standard deviations.


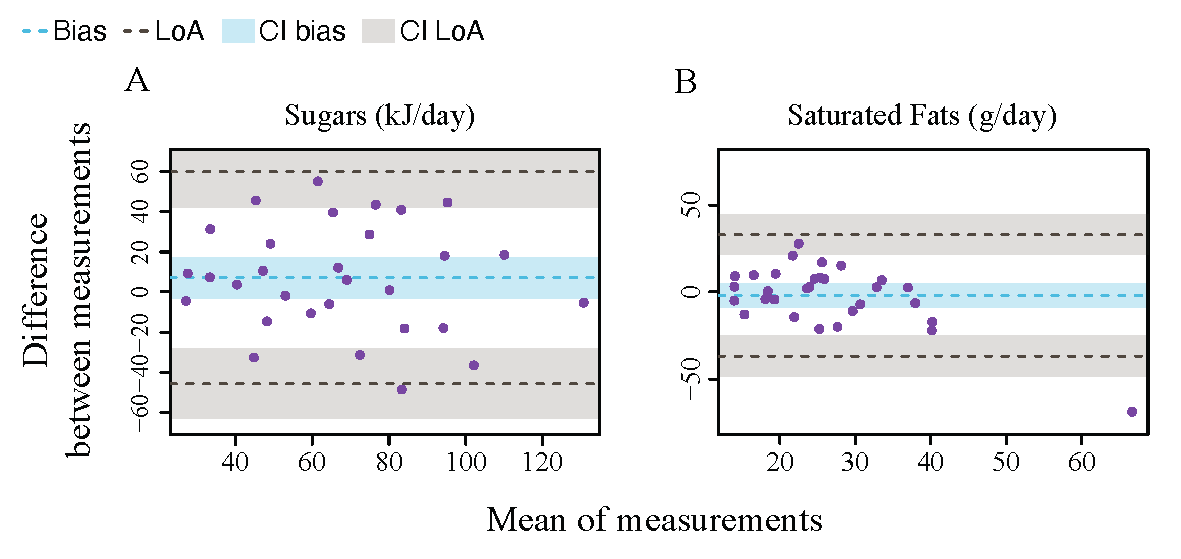


Figure S3

Measurement differences of total daily energy intake estimated with the SNAQ app and the 24-hour dietary recall (24HR) in relation to the doubly labelled water (DLW) technique. **A**, Absolute values of energy estimates with SNAQ, 24HR, and DLW. **B**, Percentage of measurement differences of SNAQ and 24HR in relation to DLW. Vertical dotted red lines mark 50-% breaks to support the interpretation of the bars on the top of the panel.

**
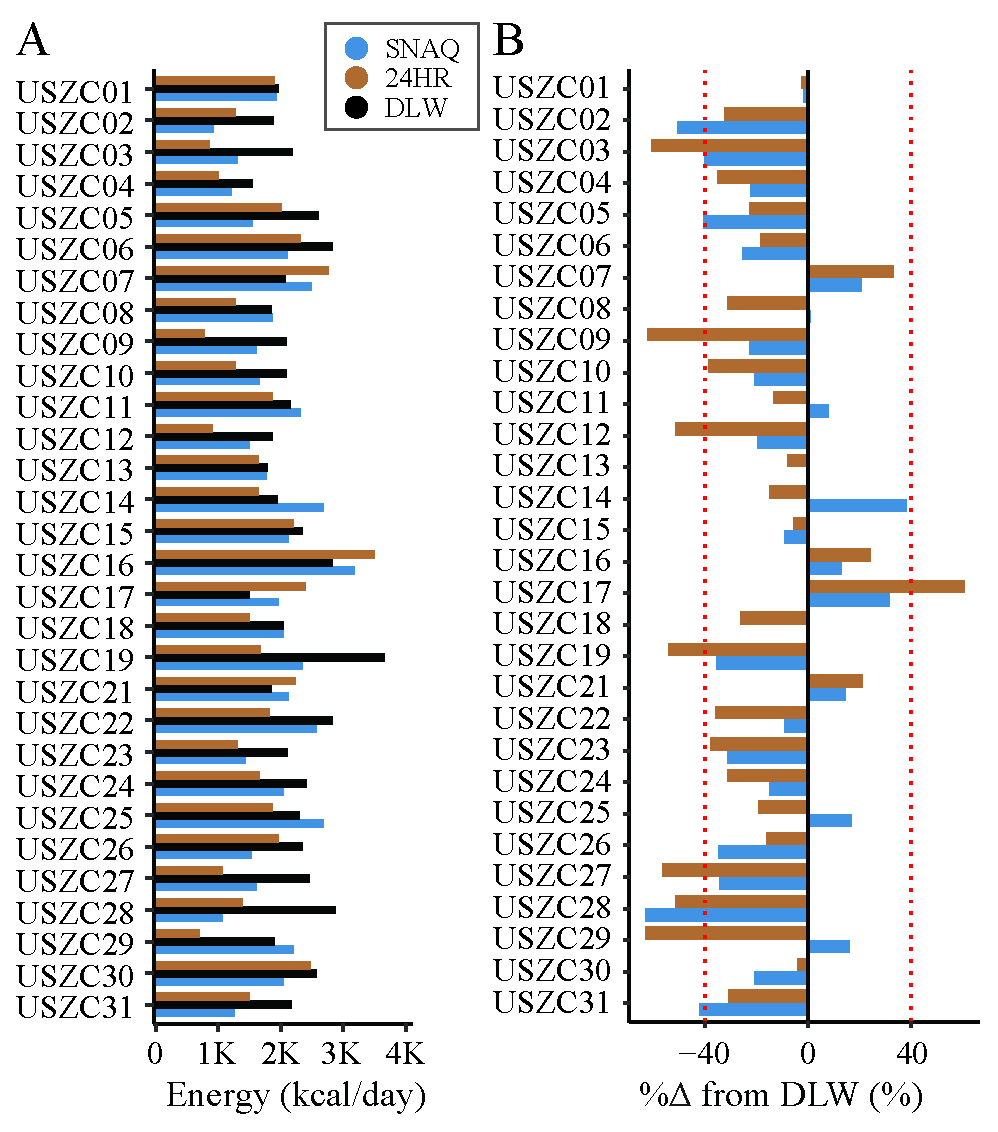
**

Figure S4

Correlation analysis of linear relationship between daily energy intake estimated with the SNAQ app and daily energy intake estimated with the 24-hour dietary recall (24HR). Abbreviations: 24HR, 24-hour dietary recall; p, p-value of the coefficient of determination; R2, coefficient of determination of the linear relationship.


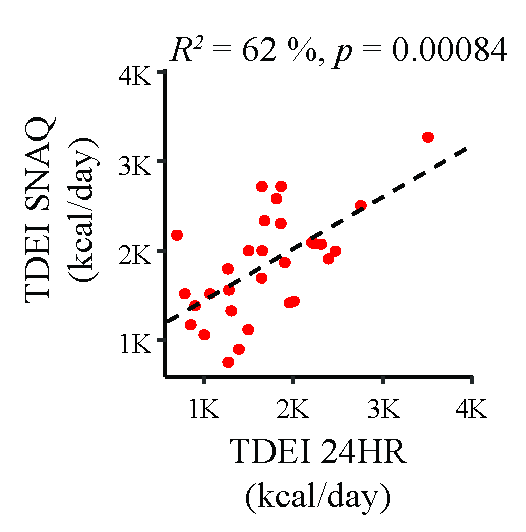


Figure S5

Absolute values of total daily energy intake (**A**), macronutrient intake (**B-G**), and eating occasions (**H**) estimated with the SNAQ app and the 24-hour dietary recall (24HR).


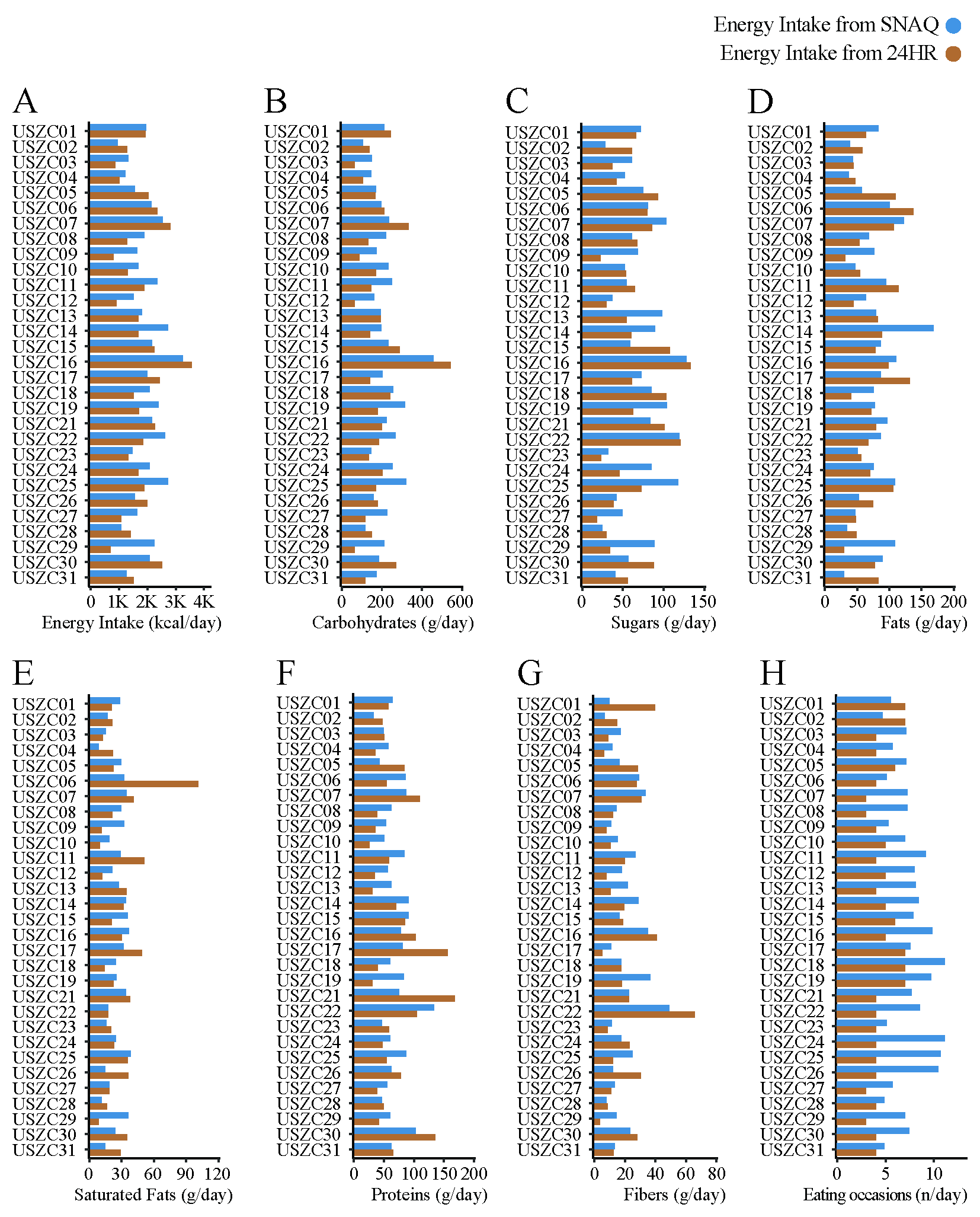


Figure S6

Boxplots of the total daily energy intake (A), of the macronutrient intake (B-G), and eating occasions (H) estimated with the SNAQ app and the 24-hour dietary recall. Paired Student’s t-test have been performed on the datasets of SNAQ and 24HR for each variable. The results of the t-tests are reported on top of each panel. Paired measurements are connected with a grey line.


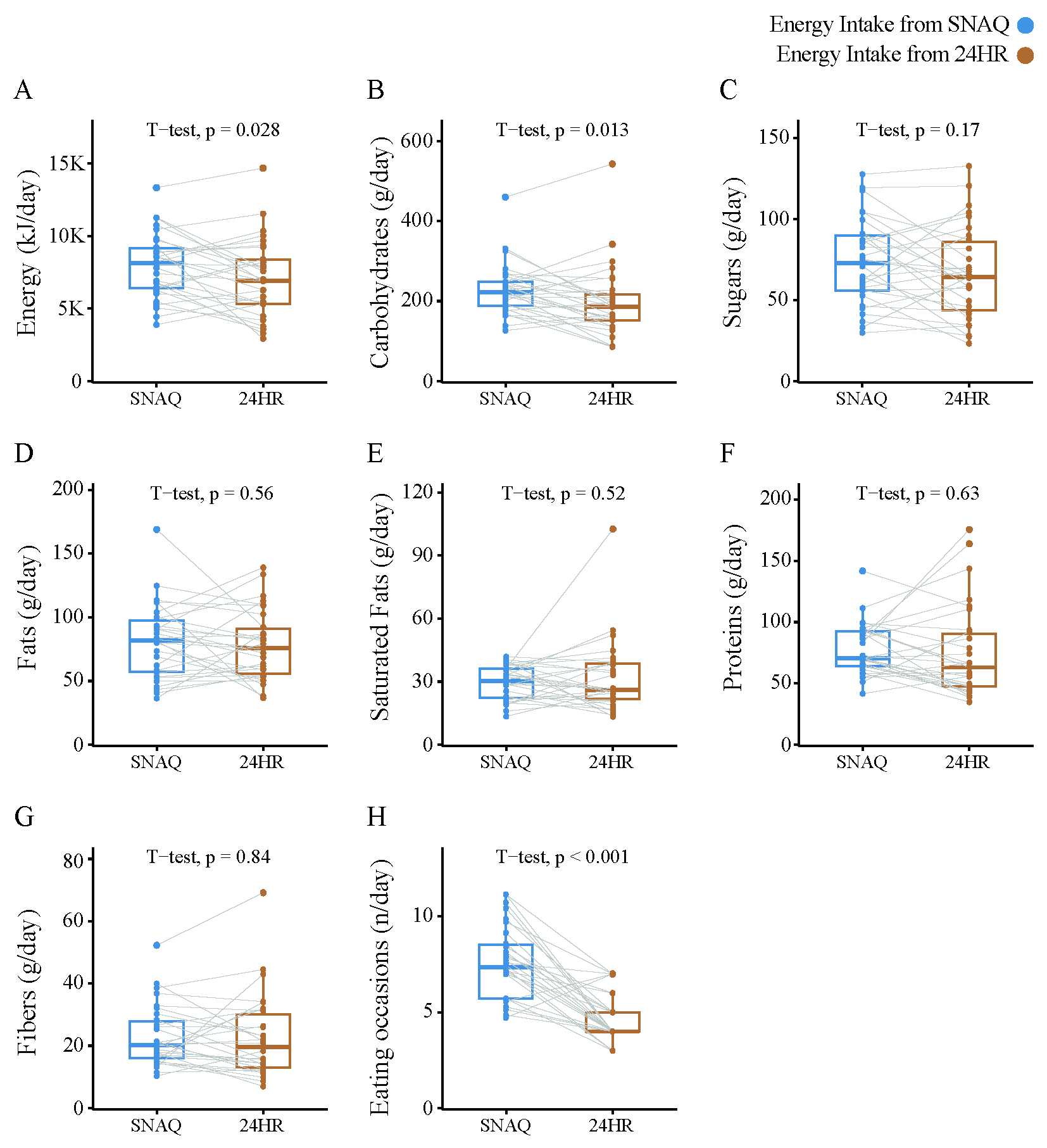


Figure S7

**
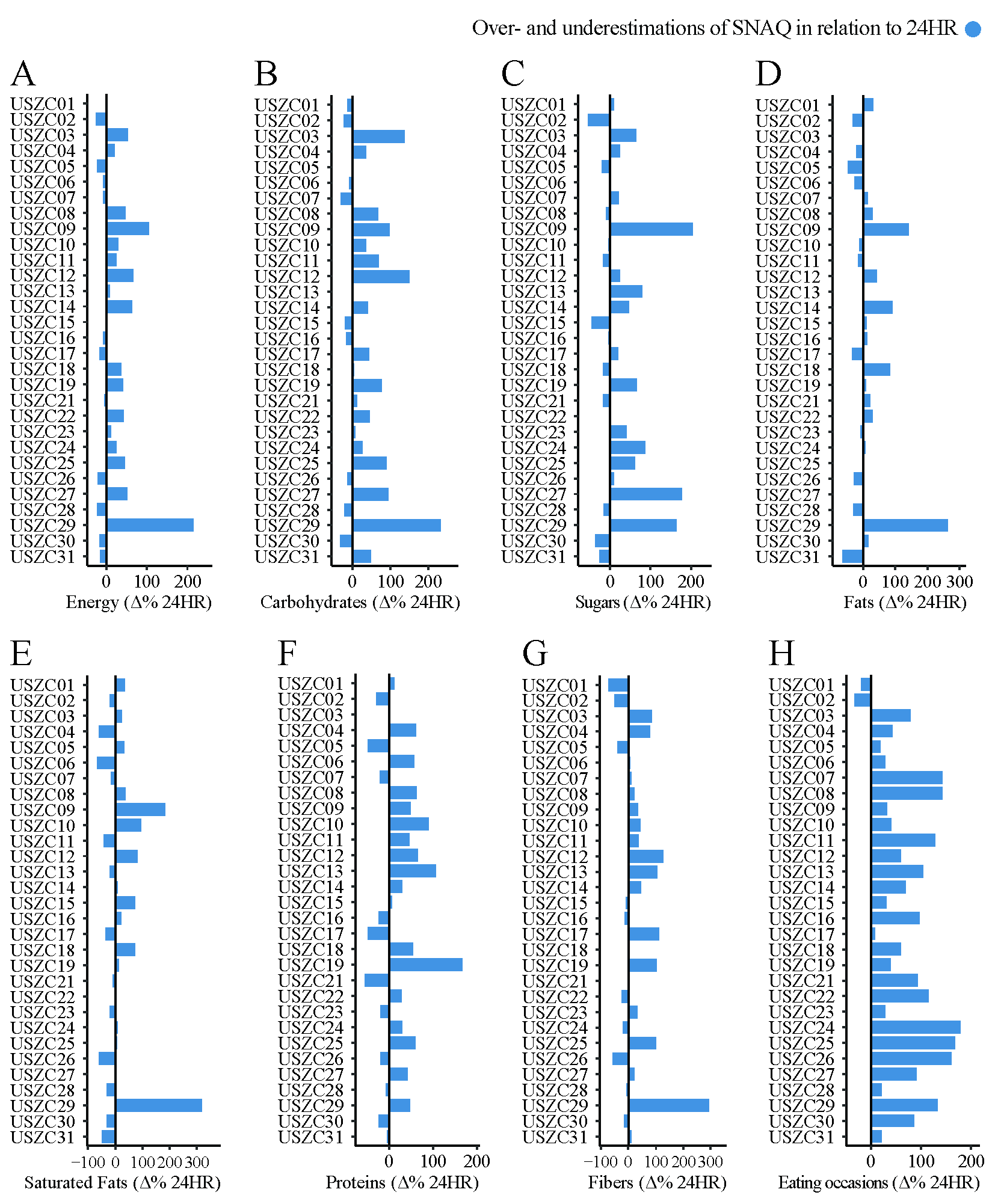
**Percentage of the measurement differences of total daily energy intake (**A**), macronutrient intake (**B-G**), and eating occasions (**H**) estimated with the SNAQ app in relation to the 24-hour dietary recall (24HR).

# Supplementary tables

Table S1

Reporting of the study according to the STROBE-nut checklist table.

**
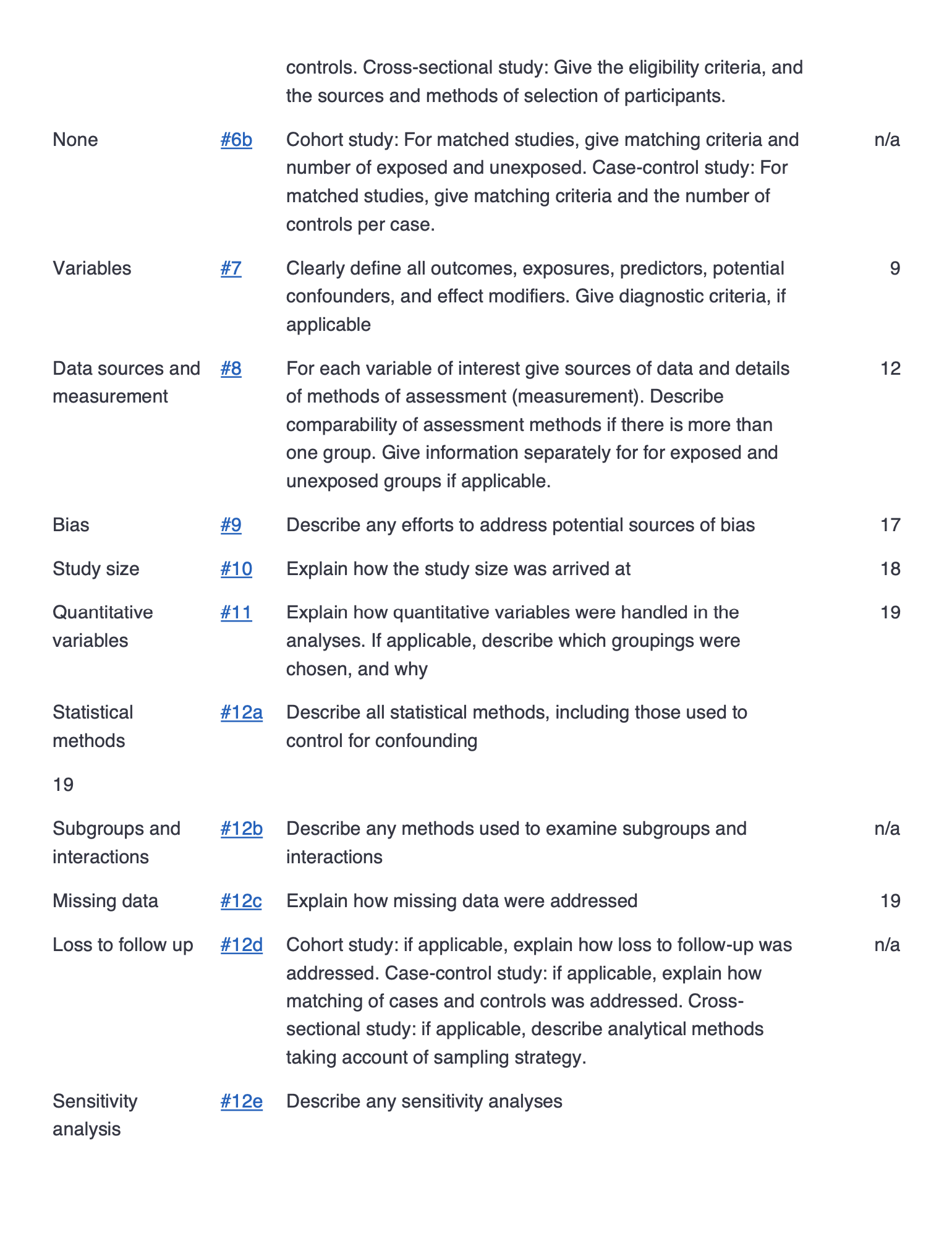
**

**
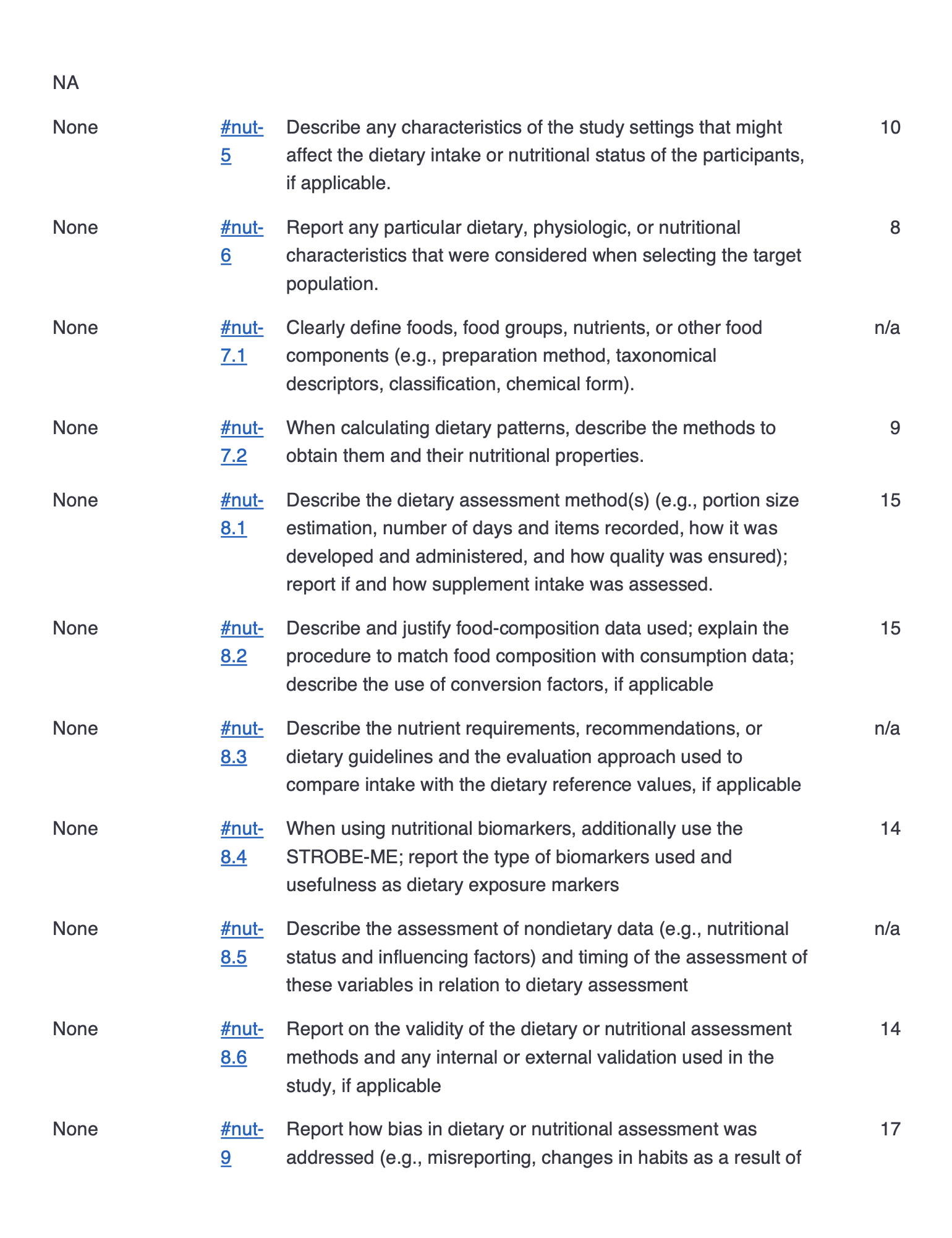
**

**
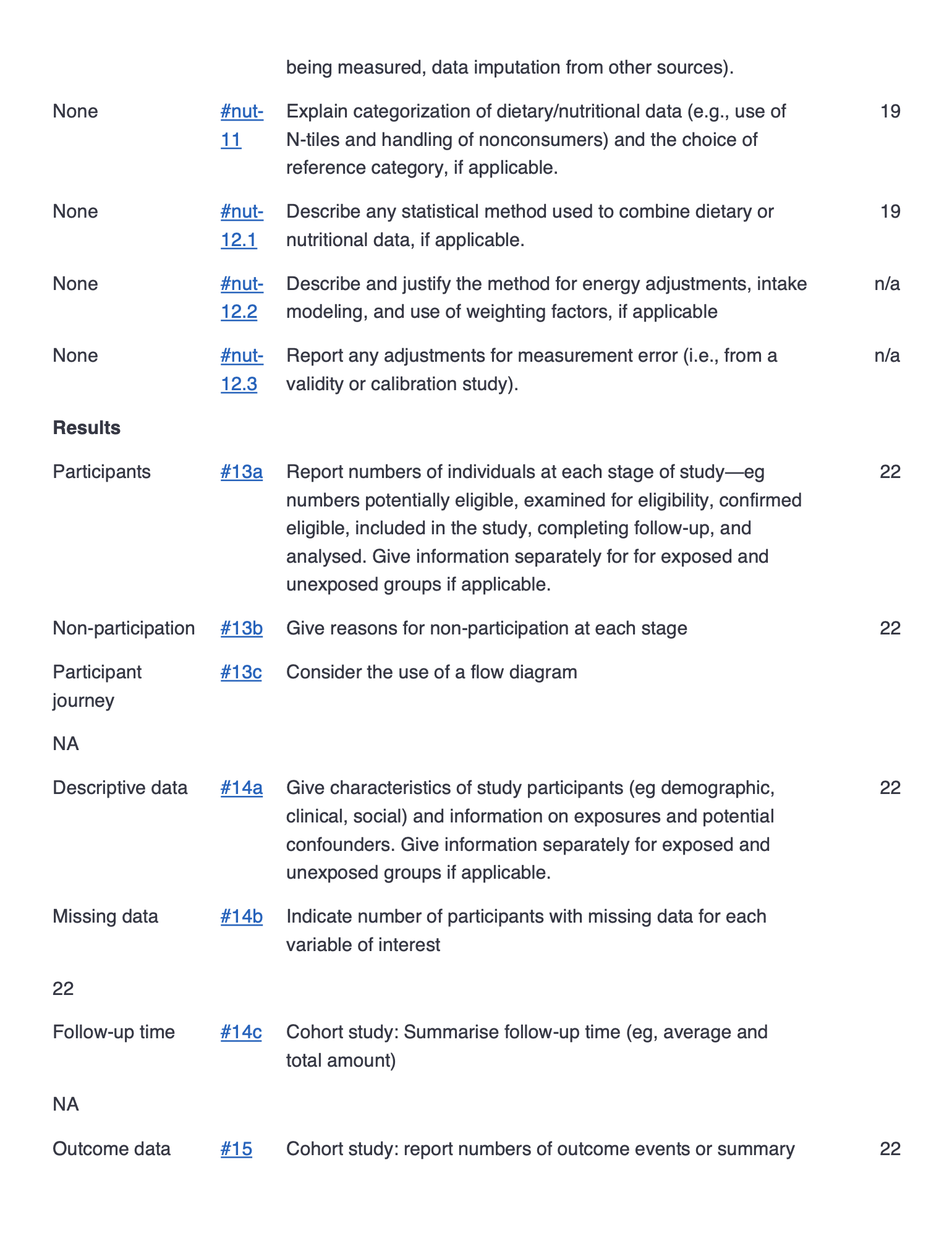
**

**
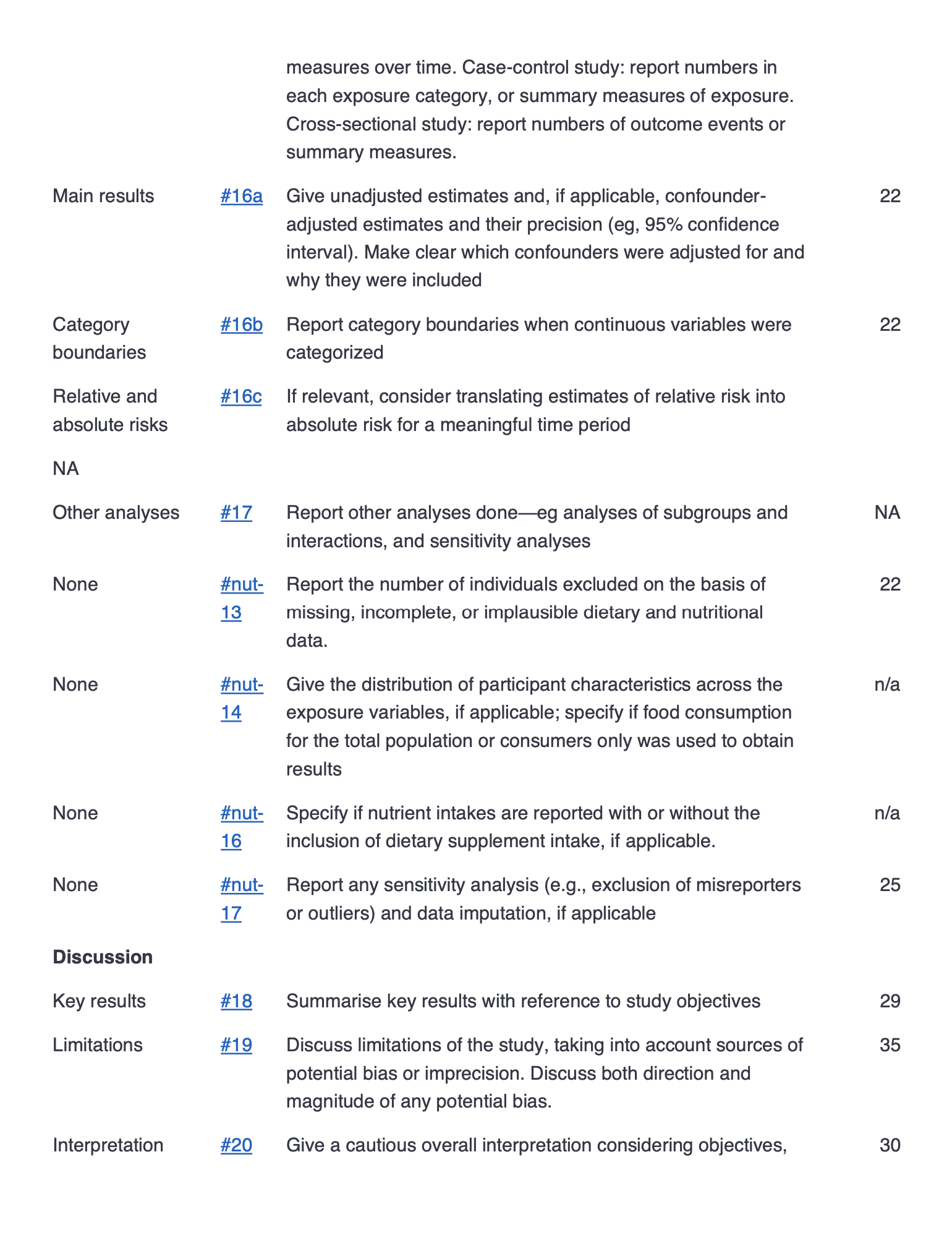
**

**
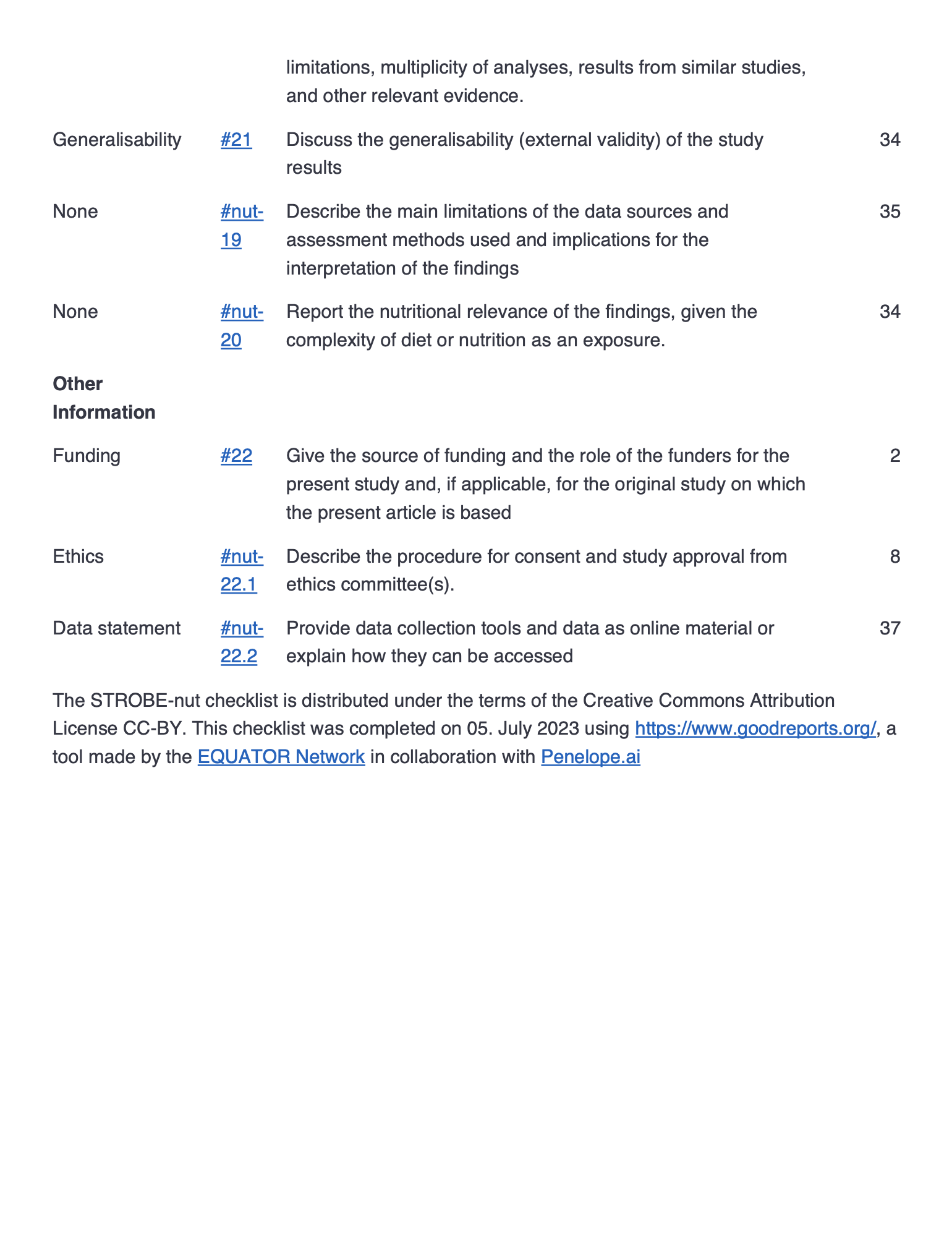
**

Table S2

Eligibility criteria.

| **Inclusion Criteria** |
| --- |
| - Adult aged 18 years or older - BMI ranging from 18.5 to 24.9 kg/m2 - No history of bariatric surgery - Independently mobile - Digital literacy - Ability to communicate fluently in English or German - Capacity to consent to participate. |
| **Exclusion Criteria** |
| - Pregnancy/lactation - Inability to understand instructions - Systemic or gastrointestinal condition which may affect food intake or preference - Diabetes Mellitus (type I and II) - Any diet for the purpose of weight-loss or weight gain - Active and significant psychiatric illness including substance misuse - Suffering from heart or kidney failure or malabsorption - Significant cognitive or communication issues - Medications with documented effect on food intake or food preference - History of significant food allergy and certain dietary restrictions - Recent travel history (overnight trip of more than 200 miles) within 2 weeks before or during the study period after dose administration of the doubly labelled water - Need for intravenous therapy during 2 weeks before and during the study period |

Table S3

Doses of doubly labelled water (DLW) for each study participant and relative DLW batch.

| **Study identifier** | **DLW dose (mL)** | **DLW batch*** |
| --- | --- | --- |
| USZC01 | 60.5973 | Batch 1 |
| USZC02 | 55.411 | Batch 1 |
| USZC03 | 60.3917 | Batch 1 |
| USZC04 | 57.2159 | Batch 1 |
| USZC05 | 71.1052 | Batch 1 |
| USZC06 | 77.0049 | Batch 1 |
| USZC07 | 67.5775 | Batch 1 |
| USZC08 | 71.1083 | Batch 1 |
| USZC09 | 60.8497 | Batch 1 |
| USZC10 | 60.1717 | Batch 1 |
| USZC11 | 60.2928 | Batch 1 |
| USZC12 | 61.3866 | Batch 1 |
| USZC13 | 73.7185 | Batch 1 |
| USZC14 | 59.8354 | Batch 1 |
| USZC15 | 73.0545 | Batch 1 |
| USZC16 | 74.1676 | Batch 1 |
| USZC17 | 70.9934 | Batch 1 |
| USZC18 | 59.3981 | Batch 1 |
| USZC19 | 60.5305 | Batch 1 |
| USZC21 | 60.0006 | Batch 2 |
| USZC22 | 61.0384 | Batch 1 |
| USZC23 | 60.4908 | Batch 3 |
| USZC24 | 66.5995 | Batch 2 |
| USZC25 | 60.501 | Batch 2 |
| USZC26 | 60.7685 | Batch 2 |
| USZC27 | 61.0006 | Batch 2 |
| USZC28 | 60.3141 | Batch 3 |
| USZC29 | 64.9196 | Batch 3 |
| USZC30 | 65.4598 | Batch 3 |
| USZC31 | 64.6201 | Batch 3 |
| * The description of the composition of the three doubly labelled water batches is available in **Table S4**. | | |

Table S4

Description of the composition of the three doubly labelled water batches.

| **DLW Batch** | **Batch date** | **Deuterium (g)** | **Lot Number D_2_O** | **18-Oxygen (g)** | **Lot Number H_2_^18^O** |
| --- | --- | --- | --- | --- | --- |
| Batch 1 | 06.05.2020 | 120.64 | I-22828E | 1803.23 | I-E2581 |
| Batch 2 | 28.01.2021 | 121.47 | I-22828E | 1802.69 | I-E2581 |
| Batch 3 | 16.08.2022 | 42.59 | PR-33098 | 632.7 | I-E2581 |

Table S5

Baseline bioelectrical impedance vector analysis of the study population.

| **Variable** | **Cohort – Normal-Weight**, N = 30* | |
| --- | --- | --- |
|  | **Bioelectrical impedance** | **Isotope Dilution** |
| **Fat mass**  *Fat mass (kg)*  *Fat mass (%)*  *Fat mass index (kg/m^2^)* | 15.8 (4.2)  26.8 (5.0)  5.8 (1.4) | 17.5 (4.3)  30.0 (5.2)  *n/a* |
| **Fat-free mass**  *Fat-free mass (kg)*  *Fat-free mass (%)*  *Fat-free mass index (kg/m^2^)* | 42.6 (3.9)  71.3 (11.6)  15.4 (1.9) | 40.3 (3.9)  *n/a*  *n/a* |
| **Body water**  *Total body water (L)*  *Total body water (%)*  *Extracellular water (L)*  *Extracellular water (%)*  *ECW/TBW* | 31.1 (3.0)  53.1 (3.4)  13.6 (1.2)  32.2 (1.5)  43.8 (2.0) | 29.5 (2.9)  *n/a*  *n/a*  *n/a*  *n/a* |
| **Visceral fat (L)** | 0.6 (0.5) |  |
| * Mean (SD). SD, standard deviation.  **Abbreviations**: *ECW*, extracellular water; *n/a*, not available; *TBW*, total body water. | | |

Table S6

Nationality of the study population.

| **Variable** | **Cohort** |
| --- | --- |
|  | **Normal-Weight**, N = 30* |
| **Nationality**  *Austria*  *Brazil*  *Ecuador*  *Germany*  *Greece*  *Israel*  *Lebanon*  *Liechtenstein*  *Mexico*  *Peru*  *Philippines*  *Spain*  *Switzerland*  *Turkey*  *U.S.A.* | 1 / 30 (3.3 %)  1 / 30 (3.3 %)  1 / 30 (3.3 %)  2 / 30 (6.6 %)  1 / 30 (3.3 %)  1 / 30 (3.3 %)  1 / 30 (3.3 %)  1 / 30 (3.3 %)  1 / 30 (3.3 %)  1 / 30 (3.3 %)  1 / 30 (3.3 %)  1 / 30 (3.3 %)  13 / 30 (43 %)  1 / 30 (3.3 %)  3 / 30 (9.9 %) |
| * Frequency (%). % percentage. | |

Table S7

Results of the doubly labelled water (DLW) analysis.

| **Study identifier** | **TBW_d_**  **(L)** | **TBW_O_**  **(L)** | **TBW**  **(L)** | **TBW**  **(mol)** | **rCO_2_**  **(ppm)** | **TDEE**  **(kcal/day)** | **PAL** | **AEE**  **(kcal/day)** | **Turnover**  **(L)** | **%TBF** |
| --- | --- | --- | --- | --- | --- | --- | --- | --- | --- | --- |
| USZC01 | 27.9 | 28.0 | 27.9 | 1549.6 | 341.4655 | 1960.454 | 1.52 | 471.41 | -3.9 | 29.4 |
| USZC02 | 25.8 | 25.9 | 25.8 | 1434.7 | 329.1987 | 1890.0268 | 1.55 | 480.01 | -3.7 | 29.4 |
| USZC03 | 28.0 | 28.2 | 28.1 | 1560.6 | 380.7693 | 2186.1086 | 1.65 | 646.50 | -3.5 | 31.0 |
| USZC04 | 24.4 | 24.6 | 24.5 | 1359.2 | 270.0075 | 1550.1925 | 1.13 | 21.17 | -2.2 | 44.3 |
| USZC05 | 32.3 | 32.3 | 32.3 | 1792.7 | 452.7403 | 2599.3152 | 1.81 | 903.38 | -4.2 | 29.7 |
| USZC06 | 36.7 | 36.7 | 36.7 | 2036.3 | 493.3261 | 2832.3304 | 1.89 | 1050.10 | -5.4 | 25.3 |
| USZC07 | 29.1 | 29.2 | 29.2 | 1619.2 | 361.1472 | 2073.4523 | 1.59 | 565.11 | -3.9 | 27.7 |
| USZC08 | 29.2 | 29.4 | 29.4 | 1629.3 | 322.0862 | 1849.1914 | 1.32 | 263.27 | -3.3 | 35.4 |
| USZC09 | 26.6 | 26.7 | 26.6 | 1478.1 | 365.1059 | 2096.1806 | 1.75 | 691.56 | -3.2 | 25.3 |
| USZC10 | 27.9 | 28.1 | 28.0 | 1555.5 | 363.7049 | 2088.1367 | 1.61 | 583.32 | -2.6 | 29.1 |
| USZC11 | 29.3 | 29.3 | 29.3 | 1625.9 | 374.0496 | 2147.5285 | 1.66 | 639.78 | -3.1 | 26.0 |
| USZC12 | 23.6 | 23.7 | 23.7 | 1314.9 | 323.97 | 1860.0073 | 1.49 | 428.01 | -2.8 | 34.0 |
| USZC13 | 29.5 | 29.7 | 29.6 | 1641.6 | 311.3148 | 1787.3498 | 1.27 | 200.61 | -3.1 | 34.3 |
| USZC14 | 28.3 | 28.4 | 28.3 | 1571.7 | 338.8784 | 1945.6007 | 1.49 | 448.04 | -3.5 | 29.8 |
| USZC15 | 31.3 | 31.2 | 31.3 | 1734.8 | 408.7091 | 2346.5192 | 1.54 | 592.87 | -4.2 | 37.9 |
| USZC16 | 32.6 | 33.0 | 32.8 | 1818.4 | 492.0052 | 2824.7464 | 2.06 | 1174.27 | -4.1 | 24.5 |
| USZC17 | 32.6 | 32.6 | 32.6 | 1807.8 | 259.7954 | 1491.5617 | 0.99 | -171.59 | -2.5 | 35.1 |
| USZC18 | 26.7 | 26.9 | 26.8 | 1487.0 | 355.3896 | 2040.396 | 1.67 | 613.36 | -2.0 | 25.8 |
| USZC19 | 31.4 | 32.7 | 32.0 | 1777.9 | 637.058 | 3657.5372 | 2.73 | 1952.78 | -3.6 | 23.9 |
| USZC21 | 26.7 | 26.7 | 26.7 | 1483.9 | 321.937 | 1848.3353 | 1.47 | 403.50 | -3.2 | 29.1 |
| USZC22 | 32.6 | 32.9 | 32.8 | 1819.0 | 493.0981 | 2831.0211 | 2.12 | 1209.92 | -5.7 | 19.5 |
| USZC23 | 32.0 | 32.1 | 32.1 | 1780.1 | 366.5656 | 2104.5608 | 1.47 | 460.10 | -3.6 | 31.2 |
| USZC24 | 29.7 | 29.9 | 29.8 | 1653.2 | 418.9813 | 2405.4947 | 1.59 | 652.95 | -3.3 | 40.6 |
| USZC25 | 34.0 | 34.2 | 34.1 | 1895.3 | 401.8549 | 2307.1673 | 1.56 | 599.45 | -4.0 | 29.3 |
| USZC26 | 29.3 | 29.5 | 29.4 | 1631.1 | 407.7768 | 2341.1666 | 1.75 | 767.05 | -4.2 | 33.6 |
| USZC27 | 28.8 | 28.8 | 28.8 | 1599.2 | 429.2006 | 2464.1669 | 1.89 | 913.75 | -2.8 | 28.4 |
| USZC28 | 30.0 | 30.2 | 30.1 | 1668.9 | 501.4032 | 2878.7034 | 2.17 | 1264.83 | -4.2 | 30.5 |
| USZC29 | 30.1 | 30.8 | 30.7 | 1705.5 | 331.1998 | 1901.5157 | 1.45 | 398.36 | -3.0 | 27.8 |
| USZC30 | 29.5 | 29.6 | 29.5 | 1638.7 | 448.7784 | 2576.5688 | 1.99 | 1023.91 | -4.3 | 27.7 |
| USZC31 | 26.6 | 26.7 | 26.7 | 1480.4 | 377.8073 | 2169.1027 | 1.74 | 706.19 | -3.3 | 26.1 |
| Abbreviations: **TBW_d_**, total body water estimated with deuterium; **TBW_O_**, total body water estimated with oxygen18; **TBW**, average estimation of total body water; **rCO_2_**, daily respiratory carbon dioxide; **TDEE**, total daily energy expenditure; **PAL**, physical activity level; **AEE**, activity-related energy expenditure; **Turnover**, water turnover during the study week; **%TBF**, percentage of the total body fat. | | | | | | | | | | |

Table S8

Results of the Shapiro–Wilk test for normality of the distribution of the energy estimates of the SNAQ app, the 24-hour dietary recall (24HR), and the doubly labelled water (DLW) technique.

| **Energy estimated with** | **Statistic*** | **p-value*** |
| --- | --- | --- |
| SNAQ | 0.99 | 0.94 |
| DLW | 0.93 | 0.047 |
| 24HR | 0.96 | 0.35 |
| * Shapiro–Wilk test.  **Abbreviation**s: *DLW*, doubly labelled water technique; *SNAQ*, the SNAQ app; *24HR*, 24-hour dietary recall. | | |

Table S9

Absolute and percentage measurement differences of total daily energy intake estimated with the SNAQ app in relation to total daily energy expenditure estimated with the doubly labelled water (DLW) technique.

| **Study identifier** | **Absolute difference**  **(kcal/day)** | **Percentage difference**  **(%)** |
| --- | --- | --- |
| USZC01 | -33.08 | -1.69 |
| USZC02 | -961.21 | -50.86 |
| USZC03 | -882.21 | -40.36 |
| USZC04 | -346.03 | -22.32 |
| USZC05 | -1058.98 | -40.74 |
| USZC06 | -719.28 | -25.4 |
| USZC07 | 425.82 | 20.54 |
| USZC08 | 15.03 | 0.81 |
| USZC09 | -481.18 | -22.96 |
| USZC10 | -435.43 | -20.85 |
| USZC11 | 172.46 | 8.03 |
| USZC12 | -365.46 | -19.65 |
| USZC13 | -15.3 | -0.86 |
| USZC14 | 745 | 38.29 |
| USZC15 | -217.23 | -9.26 |
| USZC16 | 360.3 | 12.76 |
| USZC17 | 472.75 | 31.69 |
| USZC18 | 5.82 | 0.29 |
| USZC19 | -1310.37 | -35.83 |
| USZC21 | 269.38 | 14.57 |
| USZC22 | -261.96 | -9.25 |
| USZC23 | -661.28 | -31.42 |
| USZC24 | -358.64 | -14.91 |
| USZC25 | 384.12 | 16.65 |
| USZC26 | -816.88 | -34.89 |
| USZC27 | -849.31 | -34.47 |
| USZC28 | -1819.56 | -63.21 |
| USZC29 | 301.91 | 15.88 |
| USZC30 | -534 | -20.73 |
| USZC31 | -914.25 | -42.15 |

Table S10

Absolute and percentage measurement differences of total daily energy intake estimated with the 24-hour dietary recall (24HR) in relation to total daily energy expenditure estimated with the doubly labelled water (DLW) technique.

| **Study identifier** | **Absolute difference**  **(kcal/day)** | **Percentage difference**  **(%)** |
| --- | --- | --- |
| USZC01 | -54.6 | -2.79 |
| USZC02 | -616.22 | -32.6 |
| USZC03 | -1332.59 | -60.96 |
| USZC04 | -545.11 | -35.16 |
| USZC05 | -590.78 | -22.73 |
| USZC06 | -520.17 | -18.37 |
| USZC07 | 682.77 | 32.93 |
| USZC08 | -579.81 | -31.35 |
| USZC09 | -1309.23 | -62.46 |
| USZC10 | -806.53 | -38.62 |
| USZC11 | -287.12 | -13.37 |
| USZC12 | -958.66 | -51.54 |
| USZC13 | -141.23 | -7.9 |
| USZC14 | -296.57 | -15.24 |
| USZC15 | -137.71 | -5.87 |
| USZC16 | 682.72 | 24.17 |
| USZC17 | 904.38 | 60.63 |
| USZC18 | -539.55 | -26.44 |
| USZC19 | -1978.66 | -54.1 |
| USZC21 | 387.64 | 20.97 |
| USZC22 | -1019.09 | -36 |
| USZC23 | -797.56 | -37.9 |
| USZC24 | -752.56 | -31.29 |
| USZC25 | -442.19 | -19.17 |
| USZC26 | -382.1 | -16.32 |
| USZC27 | -1395.35 | -56.63 |
| USZC28 | -1485.97 | -51.62 |
| USZC29 | -1201.29 | -63.18 |
| USZC30 | -104.63 | -4.06 |
| USZC31 | -672.87 | -31.02 |

Table S11

Classification of the study participants as over- and under-reporters for their total daily energy intake estimated with the SNAQ app according to the Goldberg cut-off points using the approach suggested by Black [1].

| **Study identifier** | **Energy Intake**  **(kcal/day)** | **BMR**  **(kcal/day)** | **TDEE:BMR**  **(PAL)** | **TDEI:BMR** | **Classification (I)^*^** | **Classification (II)^§^** |
| --- | --- | --- | --- | --- | --- | --- |
| USZC01 | 1927.4 | 1340.7 | 1.52 | 1.44 | under-reporter | under-reporter |
| USZC02 | 928.8 | 1221.0 | 1.55 | 0.76 | under-reporter | under-reporter |
| USZC03 | 1303.9 | 1338.9 | 1.65 | 0.97 | under-reporter | under-reporter |
| USZC04 | 1204.2 | 1365.1 | 1.13 | 0.88 | under-reporter | under-reporter |
| USZC05 | 1540.3 | 1496.8 | 1.81 | 1.03 | under-reporter | under-reporter |
| USZC06 | 2113.0 | 1568.1 | 1.89 | 1.35 | under-reporter | under-reporter |
| USZC07 | 2499.3 | 1306.6 | 1.59 | 1.91 | over-reporter | over-reporter |
| USZC08 | 1864.2 | 1412.0 | 1.32 | 1.32 | under-reporter | under-reporter |
| USZC09 | 1615.0 | 1227.0 | 1.75 | 1.32 | under-reporter | under-reporter |
| USZC10 | 1652.7 | 1353.8 | 1.61 | 1.22 | under-reporter | under-reporter |
| USZC11 | 2320.0 | 1309.2 | 1.66 | 1.77 | NA | NA |
| USZC12 | 1494.6 | 1237.2 | 1.49 | 1.21 | under-reporter | under-reporter |
| USZC13 | 1772.1 | 1477.3 | 1.27 | 1.20 | under-reporter | under-reporter |
| USZC14 | 2690.6 | 1378.2 | 1.49 | 1.95 | over-reporter | over-reporter |
| USZC15 | 2129.3 | 1571.7 | 1.54 | 1.35 | under-reporter | under-reporter |
| USZC16 | 3185.0 | 1447.9 | 2.06 | 2.20 | over-reporter | over-reporter |
| USZC17 | 1964.3 | 1537.6 | 0.99 | 1.28 | under-reporter | under-reporter |
| USZC18 | 2046.2 | 1259.3 | 1.67 | 1.62 | NA | NA |
| USZC19 | 2347.2 | 1344.6 | 2.73 | 1.75 | NA | NA |
| USZC21 | 2117.7 | 1327.8 | 1.47 | 1.59 | NA | NA |
| USZC22 | 2569.1 | 1318.3 | 2.12 | 1.95 | over-reporter | over-reporter |
| USZC23 | 1443.3 | 1474.2 | 1.47 | 0.98 | under-reporter | under-reporter |
| USZC24 | 2046.9 | 1578.5 | 1.59 | 1.30 | under-reporter | under-reporter |
| USZC25 | 2691.3 | 1485.1 | 1.56 | 1.81 | NA | over-reporter |
| USZC26 | 1524.3 | 1369.3 | 1.75 | 1.11 | under-reporter | under-reporter |
| USZC27 | 1614.9 | 1338.1 | 1.89 | 1.21 | under-reporter | under-reporter |
| USZC28 | 1059.1 | 1374.2 | 2.17 | 0.77 | under-reporter | under-reporter |
| USZC29 | 2203.4 | 1442.0 | 1.45 | 1.53 | NA | under-reporter |
| USZC30 | 2042.6 | 1365.4 | 1.99 | 1.50 | under-reporter | under-reporter |
| USZC31 | 1254.9 | 1246.3 | 1.74 | 1.01 | under-reporter | under-reporter |
| * Classification with an S factor specific for the study population.  § Classification with an S factor according to Black [1].  Abbreviations: *BMR*, basal metabolic rate; *TDEE*, total daily energy expenditure; *TDEI*, total daily energy intake; *NA*, plausible total daily energy intake in relation to the BMR; PAL, physical activity level. | | | | | | |

1. Black, A.E., *Critical evaluation of energy intake using the Goldberg cut-off for energy intake: basal metabolic rate. A practical guide to its calculation, use and limitations.* International journal of obesity, 2000. **24**(9): p. 1119-1130.

Table S12

Classification of the study participants as over- and under-reporters for their total daily energy intake estimated with the 24-hour dietary recall (24HR) according to the Goldberg cut-off points using the approach suggested by Black [1].

| **Study identifier** | **Energy Intake**  **(kcal/day)** | **BMR**  **(kcal/day)** | **TDEE:BMR**  **(PAL)** | **TDEI:BMR** | **Classification (I)^*^** | **Classification (II)^§^** |
| --- | --- | --- | --- | --- | --- | --- |
| USZC01 | 1905.9 | 1340.7 | 1.52 | 1.42 | under-reporter | under-reporter |
| USZC02 | 1273.8 | 1221.0 | 1.55 | 1.04 | under-reporter | under-reporter |
| USZC03 | 853.5 | 1338.9 | 1.65 | 0.64 | under-reporter | under-reporter |
| USZC04 | 1005.1 | 1365.1 | 1.13 | 0.74 | under-reporter | under-reporter |
| USZC05 | 2008.5 | 1496.8 | 1.81 | 1.34 | under-reporter | under-reporter |
| USZC06 | 2312.2 | 1568.1 | 1.89 | 1.47 | under-reporter | under-reporter |
| USZC07 | 2756.2 | 1306.6 | 1.59 | 2.11 | over-reporter | over-reporter |
| USZC08 | 1269.4 | 1412.0 | 1.32 | 0.90 | under-reporter | under-reporter |
| USZC09 | 787.0 | 1227.0 | 1.75 | 0.64 | under-reporter | under-reporter |
| USZC10 | 1281.6 | 1353.8 | 1.61 | 0.95 | under-reporter | under-reporter |
| USZC11 | 1860.4 | 1309.2 | 1.66 | 1.42 | under-reporter | under-reporter |
| USZC12 | 901.4 | 1237.2 | 1.49 | 0.73 | under-reporter | under-reporter |
| USZC13 | 1646.1 | 1477.3 | 1.27 | 1.11 | under-reporter | under-reporter |
| USZC14 | 1649.0 | 1378.2 | 1.49 | 1.20 | under-reporter | under-reporter |
| USZC15 | 2208.8 | 1571.7 | 1.54 | 1.41 | under-reporter | under-reporter |
| USZC16 | 3507.5 | 1447.9 | 2.06 | 2.42 | over-reporter | over-reporter |
| USZC17 | 2395.9 | 1537.6 | 0.99 | 1.56 | NA | NA |
| USZC18 | 1500.8 | 1259.3 | 1.67 | 1.19 | under-reporter | under-reporter |
| USZC19 | 1678.9 | 1344.6 | 2.73 | 1.25 | under-reporter | under-reporter |
| USZC21 | 1811.9 | 1327.8 | 1.47 | 1.36 | under-reporter | under-reporter |
| USZC22 | 2236.0 | 1318.3 | 2.12 | 1.70 | NA | NA |
| USZC23 | 1307.0 | 1474.2 | 1.47 | 0.89 | under-reporter | under-reporter |
| USZC24 | 1652.9 | 1578.5 | 1.59 | 1.05 | under-reporter | under-reporter |
| USZC25 | 1865.0 | 1485.1 | 1.56 | 1.26 | under-reporter | under-reporter |
| USZC26 | 1959.1 | 1369.3 | 1.75 | 1.43 | under-reporter | under-reporter |
| USZC27 | 1068.8 | 1338.1 | 1.89 | 0.80 | under-reporter | under-reporter |
| USZC28 | 1392.7 | 1374.2 | 2.17 | 1.01 | under-reporter | under-reporter |
| USZC29 | 700.2 | 1442.0 | 1.45 | 0.49 | under-reporter | under-reporter |
| USZC30 | 2471.9 | 1365.4 | 1.99 | 1.81 | NA | over-reporter |
| USZC31 | 1496.2 | 1246.3 | 1.74 | 1.20 | under-reporter | under-reporter |
| * Classification with an S factor specific for the study population.  § Classification with an S factor according to Black .[1]  Abbreviations: *BMR*, basal metabolic rate; *TDEI*, total daily energy intake; *NA*, plausible total daily energy intake in relation to the BMR. | | | | | | |

1. Black, A.E., *Critical evaluation of energy intake using the Goldberg cut-off for energy intake: basal metabolic rate. A practical guide to its calculation, use and limitations.* International journal of obesity, 2000. **24**(9): p. 1119-1130.

Table S13

Daily energy intake estimated with the SNAQ app. The energy intake from day 1 and day 8 have been excluded from the calculation of the intraclass correlation coefficient because the dietary intake of only half of the day has been recorded in both these days.

| **Study identifier** | **Day 2** | **Day3** | **Day 4** | **Day 5** | **Day 6** | **Day 7** |
| --- | --- | --- | --- | --- | --- | --- |
| USZC01 | 1581.2 | 1827.7 | 1086.6 | 1696.4 | 1624.2 | 3534.9 |
| USZC02 | 887.2 | 1980.2 | 250.5 | 111.5 | 1021.7 | 1038.2 |
| USZC03 | 1183.4 | 1897.4 | 947.8 | 2021.7 | 1248.1 | 481.5 |
| USZC04 | 1431.5 | 785.8 | 701.7 | 1367.6 | 899.7 | 1758.3 |
| USZC05 | 1200.2 | 2111.4 | 228.8 | 1946.4 | 1655.7 | 1307.2 |
| USZC06 | 2768.1 | 2669.1 | 1043.9 | 2000.5 | 2744.7 | 979.2 |
| USZC07 | 5120.4 | 1753.4 | 2363.8 | 1308.0 | 2753.6 | 1748.3 |
| USZC08 | 1381.6 | 3136.6 | 2738.9 | 1472.4 | 1499.1 | 1065.8 |
| USZC09 | 446.6 | 1006.1 | 1656.1 | 2849.4 | 2054.6 | 1092.1 |
| USZC10 | 2780.8 | 928.4 | 1580.6 | 2190.7 | 928.8 | 2075.8 |
| USZC11 | 2328.8 | 134.9 | 5208.1 | 193.4 | 1659.5 | 2297.2 |
| USZC12 | 1004.5 | 1504.8 | 1135.2 | 1673.2 | 1507.8 | 1756.0 |
| USZC13 | 2748.3 | 1627.5 | 1259.1 | 1682.5 | 1437.0 | 1535.8 |
| USZC14 | 2797.9 | 2542.8 | 1669.2 | 2685.6 | 3037.9 | 3634.2 |
| USZC15 | 2156.7 | 2332.7 | 1359.1 | 1430.6 | 1303.0 | 1497.2 |
| USZC16 | 4043.0 | 2897.8 | 2076.6 | 3463.3 | 3734.6 | 3669.0 |
| USZC17 | 1532.8 | 1488.5 | 1763.9 | 1675.3 | 2229.8 | 2721.5 |
| USZC18 | 2131.2 | 1997.9 | 2245.3 | 1575.7 | 1452.2 | 2897.3 |
| USZC19 | 1117.9 | 619.1 | 422.0 | 3698.0 | 2155.4 | 3232.4 |
| USZC21 | 1337.0 | 2629.0 | 676.0 | 1891.0 | 2123.0 | 2004.0 |
| USZC22 | 2211.1 | 3116.3 | 3159.8 | 3184.1 | 2314.9 | 1594.3 |
| USZC23 | 1380.0 | 1986.0 | 1418.0 | 1110.0 | 873.0 | 1720.0 |
| USZC24 | 2129.0 | 1999.0 | 2248.0 | 1576.0 | 1455.0 | 2899.0 |
| USZC25 | 3421.0 | 2799.0 | 3530.0 | 2319.0 | 2363.0 | 2714.0 |
| USZC26 | 697.0 | 1292.0 | 1666.0 | 1444.0 | 1320.0 | 1583.0 |
| USZC27 | 1749.0 | 2429.0 | 1144.0 | 1736.0 | 1278.0 | 1023.0 |
| USZC28 | 469.0 | 784.0 | 1362.0 | 1229.0 | 1266.0 | 930.0 |
| USZC29 | 3305.0 | 1843.0 | 2432.0 | 1018.0 | 2717.0 | 2065.0 |
| USZC30 | 2037.0 | 1489.0 | 2004.0 | 2626.0 | 1491.0 | 2094.0 |
| USZC31 | 1163.0 | 1536.0 | 919.0 | 1028.0 | 610.0 | 2137.0 |

Table S14

Absolute and percentage measurement differences of total daily energy intake estimated with the SNAQ app in relation to total daily energy intake estimated with 24-hour dietary recall (24HR).

| **Study identifier** | **Absolute difference**  **(kcal/day)** | **Percentage difference**  **(%)** |
| --- | --- | --- |
| USZC01 | 21.52 | 1.13 |
| USZC02 | -344.99 | -27.08 |
| USZC03 | 450.38 | 52.77 |
| USZC04 | 199.08 | 19.81 |
| USZC05 | -468.2 | -23.31 |
| USZC06 | -199.12 | -8.61 |
| USZC07 | -256.95 | -9.32 |
| USZC08 | 594.83 | 46.86 |
| USZC09 | 828.05 | 105.22 |
| USZC10 | 371.1 | 28.96 |
| USZC11 | 459.58 | 24.7 |
| USZC12 | 593.2 | 65.81 |
| USZC13 | 125.94 | 7.65 |
| USZC14 | 1041.57 | 63.16 |
| USZC15 | -79.52 | -3.6 |
| USZC16 | -322.42 | -9.19 |
| USZC17 | -431.62 | -18.01 |
| USZC18 | 545.36 | 36.34 |
| USZC19 | 668.29 | 39.81 |
| USZC21 | -118.26 | -5.29 |
| USZC22 | 757.13 | 41.79 |
| USZC23 | 136.29 | 10.43 |
| USZC24 | 393.93 | 23.83 |
| USZC25 | 826.31 | 44.31 |
| USZC26 | -434.78 | -22.19 |
| USZC27 | 546.04 | 51.09 |
| USZC28 | -333.59 | -23.95 |
| USZC29 | 1503.2 | 214.67 |
| USZC30 | -429.37 | -17.37 |
| USZC31 | -241.38 | -16.13 |

Table S15

Dilution spaces of the isotopes deuterium and 18-oxygen for each study participant.

| **Study identifier** | **N_d_** | **N_O_** | **N_d_/N_O_** |
| --- | --- | --- | --- |
| USZC01 | 29.0241191295588 | 28.1484323844123 | 1.03110960970002 |
| USZC02 | 26.862899928952 | 26.0690742906755 | 1.03045085642187 |
| USZC03 | 29.1990880061491 | 28.3776417472762 | 1.028946952893 |
| USZC04 | 25.4058361076946 | 24.7408382189711 | 1.02687855127776 |
| USZC05 | 33.5910402495281 | 32.5493803164688 | 1.03200245052076 |
| USZC06 | 38.1969742740146 | 36.9335507765737 | 1.03420801604167 |
| USZC07 | 30.3127314778922 | 29.4277407854844 | 1.03007334809896 |
| USZC08 | 30.4953646750074 | 29.6177951978688 | 1.02962980435497 |
| USZC09 | 27.6839360929437 | 26.85091920605 | 1.03102377540602 |
| USZC10 | 29.0946988170236 | 28.2939072108817 | 1.02830261653766 |
| USZC11 | 30.4641560512914 | 29.5242512151099 | 1.03183501011875 |
| USZC12 | 24.6154638154826 | 23.8972537638436 | 1.03005408314849 |
| USZC13 | 30.689774207146 | 29.8735683076693 | 1.02732200891004 |
| USZC14 | 29.4210854454312 | 28.5638392502048 | 1.03001158869847 |
| USZC15 | 32.5709885759681 | 31.4368356150493 | 1.03607719857071 |
| USZC16 | 33.9291068966025 | 33.1557769870566 | 1.02332413774673 |
| USZC17 | 33.8956500717667 | 32.8049833087197 | 1.03324698423965 |
| USZC18 | 27.8146746854459 | 27.0472808824967 | 1.02837230870944 |
| USZC19 | 32.6591557784154 | 32.914568375669 | 0.992240135300014 |
| USZC21 | 27.8361019929749 | 26.9133739991035 | 1.03428511021703 |
| USZC22 | 33.9564416186253 | 33.1510058529374 | 1.02429596764759 |
| USZC23 | 33.3430702697972 | 32.3347045954398 | 1.03118524467669 |
| USZC24 | 30.9096636455692 | 30.0818668375768 | 1.02751813284933 |
| USZC25 | 35.4351750151735 | 34.488353229437 | 1.02745337765006 |
| USZC26 | 30.5248871095422 | 29.6543095311675 | 1.02935754000476 |
| USZC27 | 29.9658931422191 | 29.0369814644877 | 1.03199064196351 |
| USZC28 | 31.1895976200495 | 30.3799164024103 | 1.02665185798783 |
| USZC29 | 31.8956301629351 | 31.025263965279 | 1.02805346631797 |
| USZC30 | 30.682768457092 | 29.7767117526618 | 1.03042836670336 |
| USZC31 | 27.7314918673651 | 26.8873993071973 | 1.03139361120515 |
| Abbreviations: **N_d_**, dilution space of the isotope deuterium (^2^H); **N_O_**, dilution space of the isotope oxygen18 (^18^O); **N_d_/N_O_**, dilution space ratio. | | | |

Table S16

Elimination rates of the isotopes deuterium and 18-oxygen for each study participant.

| **Study identifier** | **k_d_** | **k_O_** | **k_d_/k_O_** |
| --- | --- | --- | --- |
| USZC01 | -0.133807473546014 | -0.160177138718205 | 1.19707169168786 |
| USZC02 | -0.137276717780391 | -0.16466204803508 | 1.19948998415375 |
| USZC03 | -0.121319474902941 | -0.149557936526098 | 1.23276115929243 |
| USZC04 | -0.0851173907231945 | -0.107619893189866 | 1.2643702100767 |
| USZC05 | -0.12455068079756 | -0.153743345095532 | 1.23438381959084 |
| USZC06 | -0.142011078471093 | -0.170820414587473 | 1.20286682156452 |
| USZC07 | -0.130256765011796 | -0.156762157369439 | 1.20348572571446 |
| USZC08 | -0.108960625213557 | -0.132220689351163 | 1.21347219779638 |
| USZC09 | -0.115868406486748 | -0.144207530578287 | 1.24458025229492 |
| USZC10 | -0.0895676549189454 | -0.115674563752673 | 1.2914769718751 |
| USZC11 | -0.101032120972293 | -0.127180099969998 | 1.25880857242299 |
| USZC12 | -0.114644247210261 | -0.142878460820639 | 1.24627675873343 |
| USZC13 | -0.102091139289469 | -0.124318539553067 | 1.21772115012425 |
| USZC14 | -0.117277671735864 | -0.142592536676213 | 1.21585408855459 |
| USZC15 | -0.127658156686853 | -0.155300697428904 | 1.21653564064737 |
| USZC16 | -0.122113068427779 | -0.152983848453204 | 1.25280488339937 |
| USZC17 | -0.074964844981394 | -0.0917222527756496 | 1.22353688316723 |
| USZC18 | -0.0709621792774828 | -0.0969110489984707 | 1.3656718266715 |
| USZC19 | -0.111655278545832 | -0.150748380478489 | 1.35012318666699 |
| USZC21 | -0.115288366140571 | -0.140662718408337 | 1.2200946471635 |
| USZC22 | -0.168551901529224 | -0.201133012493568 | 1.19330016848664 |
| USZC23 | -0.108052229196614 | -0.132087026662228 | 1.22243684970053 |
| USZC24 | -0.105294619723684 | -0.13388603516386 | 1.27153728761457 |
| USZC25 | -0.111120739257809 | -0.135863812486977 | 1.22266836410944 |
| USZC26 | -0.137925735920183 | -0.16734630743694 | 1.21330733760799 |
| USZC27 | -0.0941539263018247 | -0.123810763235789 | 1.31498247708645 |
| USZC28 | -0.134726433861555 | -0.168974906724705 | 1.25420752172765 |
| USZC29 | -0.0969424926284874 | -0.119432040984286 | 1.23198855059344 |
| USZC30 | -0.138702674672257 | -0.170488728487614 | 1.22916684116197 |
| USZC31 | -0.119013302549806 | -0.148268167885781 | 1.24581172616173 |
| Abbreviations: **k_d_**, elimination rate of the isotope deuterium (^2^H); **k_O_**, elimination rate of the isotope oxygen18 (^18^O); **k_d_/k_O_**, elimination rate ratio. | | | |

Table S17

Results of the Bland-Altman plot for agreement between bioelectrical impedance (BIA) and isotope dilution of doubly labelled water (DLW) for measurements of body composition in terms of Total Body Water (TBW), Fat-Free Mass (FFM), and Fat Mass (FM). TBW, FFM, and FM were calculated in kg. DLW was selected as reference method for the analysis.

| **Agreement between:** | **Bias** | **lCI bias** | **uCI bias** | **SD bias** | **SE bias** | **lLoA** | **uLoA** | **SE LoA** | **lCI lLoA** | **uCI lLoA** | **lCI uLoA** | **uCI uLoA** |
| --- | --- | --- | --- | --- | --- | --- | --- | --- | --- | --- | --- | --- |
| TBW | 1.5 | 0.9 | 2.0 | 1.4 | 0.3 | −1.4 | 4.3 | 0.5 | −2.3 | −0.4 | 3.3 | 5.2 |
| FFM | 2.1 | 1.4 | 2.8 | 1.9 | 0.4 | −1.6 | 5.8 | 0.6 | −2.9 | −0.4 | 4.6 | 7.1 |
| FM | −1.8 | −2.5 | −1.1 | 1.9 | 0.3 | −5.5 | 1.9 | 0.6 | −6.7 | −4.2 | 0.7 | 3.1 |
| Abbreviations: **Bias**, bias of agreement; **lCI**: lower 95 % confidence interval; **lLoA**, lower limit of agreement; **SD**, standard deviation; **SE**, standard error; **uCI**, upper 95 % confidence interval; **uLoA**, upper limit of agreement. | | | | | | | | | | | | |
